# Analysis approaches to address treatment nonadherence in pragmatic trials with point-treatment settings: a simulation study

**DOI:** 10.1186/s12874-022-01518-8

**Published:** 2022-02-16

**Authors:** Md. Belal Hossain, Lucy Mosquera, Mohammad Ehsanul Karim

**Affiliations:** 1 School of Population and Public Health, University of British Columbia, Vancouver, BC Canada; 2 Department of Statistics, University of British Columbia, Vancouver, BC Canada; 3 Centre for Health Evaluation and Outcome Sciences, St. Paul’s Hospital, Vancouver, BC Canada

**Keywords:** Pragmatic trials, Nonadherence, Simulation, Unmeasured confounding

## Abstract

**Background:**

Two-stage least square [2SLS] and two-stage residual inclusion [2SRI] are popularly used instrumental variable (IV) methods to address medication nonadherence in pragmatic trials with point treatment settings. These methods require assumptions, e.g., exclusion restriction, although they are known to handle unmeasured confounding. The newer IV-method, nonparametric causal bound [NPCB], showed promise in reducing uncertainty compared to usual IV-methods. The inverse probability-weighted per-protocol [IP-weighted PP] method is useful in the same setting but requires different assumptions, e.g., no unmeasured confounding. Although all of these methods are aimed to address the same nonadherence problem, comprehensive simulations to compare performances of them are absent in the literature.

**Methods:**

We performed extensive simulations to compare the performances of the above methods in addressing nonadherence when: (1) exclusion restriction satisfied and no unmeasured confounding, (2) exclusion restriction is met but unmeasured confounding present, and (3) exclusion restriction is violated. Our simulations varied parameters such as, levels of adherence rates, unmeasured confounding, and exclusion restriction violations. Risk differences were estimated, and we compared performances in terms of bias, standard error (SE), mean squared error (MSE), and 95% confidence interval coverage probability.

**Results:**

For setting (1), 2SLS and 2SRI have small bias and nominal coverage. IP-weighted PP outperforms these IV-methods in terms of smaller MSE but produces high MSE when nonadherence is very high. For setting (2), IP-weighted-PP generally performs poorly compared to 2SLS and 2SRI in term of bias, and both-stages adjusted IV-methods improve precision than naive IV-methods. For setting (3), IV-methods perform worst in all scenarios, and IP-weighted-PP produces unbiased estimates and small MSE when confounders are adjusted. NPCB produces larger uncertainty bound width in almost all scenarios. We also analyze a two-arm trial to estimate vitamin-A supplementation effect on childhood mortality after addressing nonadherence.

**Conclusions:**

Understanding finite sample characteristics of these methods will guide future researchers in determining suitable analysis strategies. Since assumptions are different and often untestable for IP-weighted PP and IV methods, we suggest analyzing data using both IP-weighted PP and IV approaches in search of a robust conclusion.

## Background

Randomization is the core principle of clinical trials, that protects against noncomparability between treatment groups at baseline. Pragmatic trials are getting popular for exploring the effectiveness of treatments in settings that mimic real-world clinical practice [1]. In this manuscript, we focus on nonadherence in a point-treatment setting, where treatment is assigned at baseline shortly after randomization. Although the treatments are randomly assigned at baseline for pragmatic trials, it is possible that some subjects may deviate from the protocol because of switching to other treatments, loss to follow-up due to side-effects, etc. In the presence of treatment nonadherence, a treatment effect estimate that is agnostic to the adherence pattern, is less useful for the patients and caregivers to make a decision about the treatment. Moreover, pragmatic trials are often unblinded [2], which often introduces selection bias [3]. Typically, those adherent and nonadherent subjects are different in terms of prognostic factors [4], and it is necessary to take into account those factors while estimating the treatment effect. An additional challenge arises when adherence depends on subject’s char measured during baseline. Inability to adjust for the unmeasured confounding could bias the treatment effect, depending on the method of estimation [4, 5].

An intention-to-treat (ITT) analysis is treated as a default analytic technique to address nonadherence in randomized and pragmatic trial settings [6]. The baseline randomization preserved by this analysis, and hence baseline confounding is not a concern [7, 8]. The per-protocol (PP) and as-treated (AT) are two other common methods that are popularly used to address nonadherence [9, 10]. In practice, all these naive methods usually produce biased estimates if the nonadherence occurs in a nonrandom fashion [4]. Typically that is the case, if some of the characteristics of the patient act as a confounder, e.g., are predictive of the nonadherence pattern as well as the outcome of interest. Particularly for PP approaches, when subjects who deviate from protocol are removed from the analysis, comparability of the subjects in both arms ensured by the process of randomization is violated. In that case, baseline confounder adjusted-PP methods are utilized to address the nonadherence in pragmatic trials, if the baseline measurements of the necessary confounders are available [7, 11–13]. As an alternative, inverse probability (IP)-weighted PP is also used to produce marginal estimates, and that can adjust for the measured confounders. However, if some of the necessary confounders are not measured, the PP based methods usually provide biased estimates [14]. In that case, the instrumental variable (IV)-based methods can still be used to get the unbiased estimate of the treatment effect [15], which is a known strength of these IV methods. Previous studies used various versions of IV-based methods to address the nonadherence in the pragmatic trial settings [10, 16, 17]. Two-Stage least squares (2SLS), two-stage residual inclusion (2SRI) are well-known IV-based methods, with a known limitation that they are usually inefficient. Newer IV-based method, nonparametric causal bound (NPCB), is proposed in the literature which uses a partial identification approach, but provides only bounds rather than point estimates [18]. This method was touted as a promising method in terms of reducing the levels of uncertainty, but the original method can not adjust for any confounders. Two previous studies analyzed data from a two-arm randomized control trial in northern Sumatra using this NPCB method [19, 20]. They reported very wide bounds from NPCB method. As a motivating example, we used the same dataset to explore the performance of two other IV methods in the same scenario, and from the simulations under various parameter spaces, we investigated the possible reasons of why the NPCB may have produced wide bounds in this study.

Both the adjusted PP methods (baseline adjusted and IP-weighted) and IV-based methods (2SLS, 2SRI, and NPCB) aim to deal with adherence adjustment but require different assumptions. For example, adjusted PP approaches assume there is no unmeasured confounding, while the IV-based methods assume the exclusion restriction (the IV is associated with the outcome only through the treatment) [21–23]. These two assumptions cannot be empirically verified given the observed data, but the violation of the assumptions can lead to biased estimates [14, 24]. Let we are interested in estimating the effect of a heart transplant on one-year mortality. No unmeasured confounding implies that all necessary confounders (e.g., variables causally associated with both heart transplant and mortality) are measured. If randomization is the instrument, the exclusion restriction assumption suggests that randomization should not directly influence any variable other than whether patients did the heart transplant versus standard care. Despite the different statistical assumptions required by these two classes of methods dealing with the same nonadherence problem, the comprehensive comparison of these adherence-adjusted methods remains largely absent in the literature. Moreover, there are two definitions of PP methods, such as (a) censoring those patients if and when deviating from the protocol and (b) excluding those patients entirely from the analysis [25]. There exist some recent simulations in the literature for the first definition [26, 27] but we could not find much explorations for the second definition. Besides, NPCB being a relatively newly proposed method, finite sample characteristics of its estimates under the above settings should be explored.

In the present study, we aimed to compare two adjusted PP approaches and three versions of the IV-based methods in the presence of nonadherence. To explore the benefits and limitations of the applications of these approaches (along with the naive approaches), and to evaluate how robust these methods are if the respective assumptions are violated, we propose comprehensive simulation studies to compare these methods under different settings, such as when (1) exclusion restriction satisfied and no unmeasured confounding, (2) exclusion restriction satisfied but unmeasured confounding present, and (3) exclusion restriction violated. Under these scenarios, we try to identify which methods are more appropriate to use.

## Methods

### Estimation methods

#### Methods

We compared the estimates of the following methods for a binary outcome of interest: naive methods (ITT, naive PP, and naive AT), two PP methods (baseline adjusted PP and IP-weighted PP), and three classes of IV-based methods (2SLS, 2SRI, and NPCB). Some earlier studies [28, 29] used the 2SLS method where confounders are adjusted only in the first stage of the model. Wang et al. [29] used the 2SRI method with confounders adjusted only in the first stage of the model. To compare whether there are any effects of considering different versions of these IV methods, we have added some variations of all these methods, such as the naive, first-stage adjusted, and both-stages adjusted of 2SLS and 2SRI approaches. A brief description of these models is provided in Table 1, and the full description can be found in Appendix A.

**Table 1:** Description of the estimation methods used for dealing with treatment nonadherence in pragmatic trials with point-treatment settings

Name of the method	Description
Naïve methods	
ITT	It models the randomization variable (*Z*) on the outcome (*Y*) without adjustment for measured confounders *L*. This method does not consider whether individuals adhered to the treatment [6].
Naïve PP	It models *Z* on *Y* among those subjects who receive the treatment according to the protocol but without adjustment for *L*. This method excludes those subjects who deviated from the protocol.
Naïve AT	It models the treatment actually received (*A*) on *Y* without adjustment for *L*. This method does not consider whether individuals randomized to the treatment groups.
Adjusted methods	
Baseline-adjusted ITT	The same as ITT but it adjusts for *L*.
Baseline-adjusted PP	The same as naïve PP but it adjusts for *L*.
IP-weighted PP	This method creates inverse probability adherence weights to generate a pseudo population to estimate the treatment effect by removing the effect of nonadherence [25]. We used a logistic model that adjusts for *L* to estimate the probabilities, and then used the marginal structural model to estimate the parameters of interest. The stabilized weights were used to prevent from extreme weights [7, 30].
IV-methods	
Naïve 2SLS	The instrument (*Z*) is modelled to the treatment (*A*) in the first stage, and then the predicted treatment is modelled to the outcome (*Y*) in the second stage [31]. There was no adjustment for *L* in either stage of the model.
First-stage adjusted 2SLS	The same as naive 2SLS except it adjusts for *L* in the first stage of the model [28, 29].
Both-stages adjusted 2SLS	The same as naive 2SLS except it adjusts for *L* in both stages of the model.
Naïve 2SRI	The instrument (*Z*) is modelled to the treatment variable (*A*) in the first stage, and then the residuals from the first stage and the treatment variable are modelled to the outcome (*Y*) in the second stage [22]. There was no adjustment for *L* in either stage of the model.
First-stage adjusted 2SRI	The same as naive 2SRI except it adjusts for *L* in the first stage of the model [29].
Both-stages adjusted 2SRI	The same as naive 2SRI except it adjusts for *L* in both stages of the model [22].
NPCB	This nonparametric method uses a constrained probability statement to provide bounds on the estimated treatment effect rather than a point estimate [18, 19].

Note: The 2SLS, 2SRI, and NPCB are IV-based methods. Whether there is any adjustment for covariates, the 2SLS/2SRI are not termed as the naive, first-stage adjusted, or both-stages adjusted 2SLS/2SRI in the literature. For comparison purposes, we termed these methods as the naive, first-stage adjusted, or both-stages adjusted 2SLS/2SRI;

Abbreviations: ITT: intention-to-treat; PP: per-protocol; AT: as-treated; IP-weighted PP: inverse probability weighted per-protocol; 2SLS: two-stage least square; 2SRI: two-stage residual inclusion model; NPCB: non-parametric causal bound.

#### Assumptions

The key assumption for the PP methods and IV-based methods is the no unmeasured confounding and exclusion restriction, respectively [21–23]. The other assumptions of all the methods are provided in Table 2 and are summarized in Appendix A.

**Table 2:** Assumptions of the estimation methods described in Table 1 that are used for addressing treatment nonadherence in pragmatic trials with point-treatment settings

Name of the method	Key assumption	Other assumptions
ITT, baseline-adjusted ITT, naïve PP, naïve AT	No unmeasured confounding	Consistency, no interference, positivity, nonadherence occurred completely at random
Baseline-adjusted PP, IP-weighted PP	No unmeasured confounding	Consistency, no interference, positivity, correct model specification
Naïve 2SLS, first-stage adjusted 2SLS, both-stages adjusted 2SLS	Exclusion restriction	Consistency, no interference, positivity, correct model specification, relevance, monotonicity
Naïve 2SRI, first-stage adjusted 2SRI, both-stages adjusted 2SRI	Exclusion restriction	Consistency, no interference, positivity, correct model specification, relevance, monotonicity, linearity of residuals
NPCB	Exclusion restriction	Consistency, no interference, positivity, relevance, monotonicity

Abbreviations: ITT: intention-to-treat; PP: per-protocol; AT: as-treated; IP-weighted PP: inverse probability weighted per-protocol; 2SLS: two-stage least square; 2SRI: two-stage residual inclusion model; NPCB: non-parametric causal bound.

**Fig. 1:**
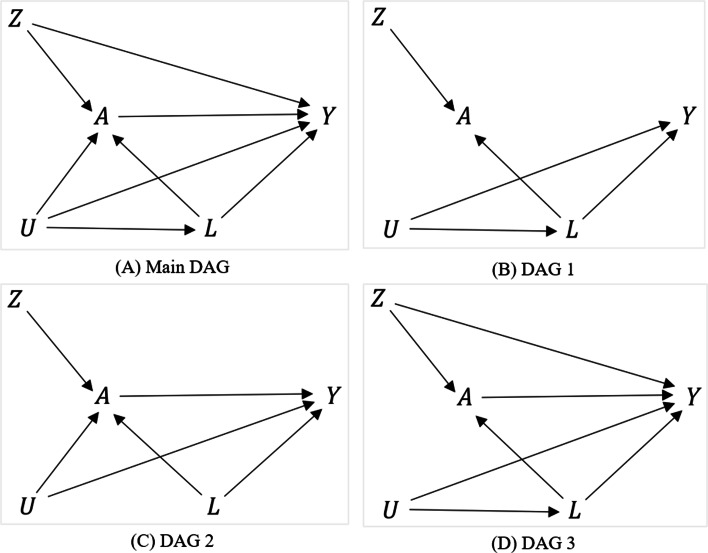
The causal diagrams representing the simulation mechanisms considered in this study. DAGs 1-3 are subsets of the main DAG and represent the simulation settings 1-3, respectively. Here, *Z* is the randomization variable, *A* is the treatment, *L* is a vector of measured confounders, *U* is unmeasured confounders, and *Y* is the outcome. Abbreviations: DAG: directed acyclic graph

Confounders, confounding, and exclusion restriction are the three important concepts to understand the simulation settings we describe below. The traditional definition of a “confounder" is that it meets all three conditions: the variable is (i) causally associated with the outcome, (ii) non-causally or causally associated with the treatment, and (iii) not in the causal pathway between the treatment and outcome [32]. Consider the causal diagram is shown in Fig. 1(A), where *Z* is the randomization, *A* is the treatment, and *Y* is the outcome. Consider two variables in the figure: *L* (measured) and *U* (unmeasured). Both *L* and *U* are (i) causally associated with *Y*, (ii) causally associated with *A*, and (iii) not in the causal pathway between *A* and *Y* (i.e., not mediators). *L* and *U* are also not colliders (i.e., not common effect of *A* and *Y*) [33], but confounders in this example. On the other hand, “confounding" is a fundamental concept in epidemiological studies to estimate causal effects. No unmeasured confounding implies that the distribution of *Y* would be the same for the treated and untreated subjects if both received the same treatment. That means, the treated and untreated subjects are comparable in terms of any measured and unmeasured factors if both had been treated [33]. To address confounding, in the causal diagram terminology, we must close all open “backdoor paths" between *A* and *Y* (e.g., adjust for those variables in the regression model that block the open backdoor paths) [33, 34]. There could be scenarios where more than one variable lies along a backdoor path. In such a scenario, adjusting for a single confounder on the path can be sufficient to block the backdoor path [33, 34]. For example, in Fig. 1(B), although we have two “confounders" (*L* and *U*) by the conventional confounder definition (*A*←*L*→*Y*, and *A*←*L*←*U*→*Y*), adjusting only for *L* is sufficient to close all open backdoor paths between *A* and *Y*. That means, *L* constitutes the minimal sufficient adjustment set [35]. In such a case, adjusting only for *L* can address “confounding" (even though there remained *U* unadjusted, which is a confounder by conventional definition), and it is certainly possible to get an unbiased effect of *A* on *Y* [33], irrespective of whether we can measure or adjust *U*. But in Fig. 1(C), adjusting only for *L* is not sufficient to close all open backdoor paths between *A* and *Y* (since a path is open through *U*), indicating the presence of confounding in the *A*-*Y* relationship. For more discussion on confounders and confounding, please see [32, 33].

The exclusion restriction assumption says the instrument is associated with the outcome only through the treatment. For example, in Figs. 1(B)-(C), the instrument *Z* is associated with *Y* only through its effect on *A*, meaning that the exclusion restriction is met [36]. As shown in Fig. 1(D), if the instrument influences *Y* through a pathway other than *A*, the exclusion restriction assumption is violated [37].

#### Target parameter

The risk difference (RD) was calculated as the target parameter of interest. The odds ratio is a widely used association measure for a binary outcome. However, since the odds ratio is a non-collapsible measure, we chose not to use this measure [38]. Our effect estimates are marginal for some methods and conditional over covariates for other methods. For example, the outcome model for the naive methods, naive and first-stage adjusted 2SLS, and IP-weighted PP do not include any covariates, and the resulting effect estimates are marginal. On the other hand, the baseline-adjusted methods, both-stages adjusted 2SLS and 2SRI, include measured covariates in the outcome model, and the resulting effect estimates are conditional on covariates. Therefore, we chose to use RD, which is a collapsible effect measure that gives equivalent and comparable marginal or conditional estimates [39].

To estimate RD, we can use the binomial regression with an identity link function as the outcome model. However, the binomial model fitting frequently shows convergence issues with adjusting for covariates in the model and requires guessing reasonable starting values which is almost impossible in the simulation settings. Naimi and Whitcomb [40] suggested to use the generalized linear model (GLM) with a Gaussian (i.e., normal) distribution in such scenarios as an alternative to the binomial regression even if the outcome variable is binary. The authors suggested using the robust sandwich standard error for valid standard errors in this setting. In our study, we followed the GLM method described by Naimi and Whitcomb as the outcome model for all the estimators other than the IP-weighted PP [40]. For the IP-weighted PP estimator, since we have to incorporate IP weights in the outcome model, we used the weighted GLM for the Gaussian family with an identity link function and robust standard error.

### Simulation setup

We followed two established simulation structures described by Young et al. [26], and by Palmer et al. [19] with modifications. Under both structures, we consider point-treatment settings (treatment is administered only once) and each individual is assigned to the treatment (*Z*=1) or to standard care (*Z*=0). We considered three data generating mechanisms, where *A* is the treatment, *Y* is the binary outcome of interest, *L*=(*L*_1_,*L*_2_) is a vector of measured baseline confounders, and *U* is unmeasured confounders. We considered one set of variables in the following causal order for all three settings: *Z*,*U*,*L*_1_,*L*_2_,*A*,*Y*. One such example of a real-world scenario could be estimating the effect of the seasonal influenza vaccine on flu in 6 months among adults. The three data-generating mechanisms considered in this study are different than each other in terms of the underlying assumption of measured and unmeasured confounding and exclusion restriction. Figure 1(A) shows the overall directed acyclic graph (DAG) of the data generating process. For simulation settings 1-3 described below, we simulated data from the following algorithms (Eqn. (1)):

1 $$ \begin{aligned} Z \sim \text{Bernoulli}(0.5) \\ U \sim f_{U} \\ L_{1} \sim \text{Normal}(\lambda_{0} + \lambda_{1} U, \sigma) \\ L_{2} \sim \text{Bernoulli}(p_{L2}) \\ A \sim \text{Bernoulli}(p_{A}) \\ Y \sim \text{Bernoulli}(p_{Y}). \end{aligned}  $$

The description of each three generating processes with parameterization is described below in detail.

#### Setting 1: exclusion restriction satisfied and no unmeasured confounding

Young et al. [26] described the simulation structure for longitudinal setting, whereas we used a simplified version of this simulation by considering the point treatment setting and having only baseline covariate measurements. Data for setting 1 are generated from our example of the influenza vaccine effect on flu. *Z* and *A* are the randomization and influenza vaccine status, receptively. In this example, physical activity (*L*_1_) and illness (*L*_2_) are two measured confounders, and smoking is an unmeasured confounder (*U*). Less physical activity can cause chronic illness, and thus, an arrow from *L*_1_ to *L*_2_ in our example could be justified [41]. Besides, smoking typically reduces the level of physical activity (*U*→*L*_1_) [42], increases illness (*U*→*L*_2_) [43], and increases the risk of flu (*U*→*Y*) [44]. In addition, people with less physical activity and having chronic illness could have influenza vaccine hesitancy and are less likely to take the vaccine (***L***→*A*) [45]. DAG 1 in Fig. 1(B) is a subset of Fig. 1(A), that shows the data generating process for setting 1. The exclusion restriction assumption is satisfied for this simulation framework. To get an unbiased effect of the influenza vaccine on flu, we must block all open backdoor paths between the vaccine and flu. As shown in Fig. 1(B), even though we have an unmeasured confounder (e.g., smoking), adjusting for measured confounders can be sufficient to block the backdoor path between vaccine status and flu status. Whether or not we measure or adjust for smoking does not impact our ability to obtain unbiased treatment effect estimates of the influenza vaccine on flu. In other words, adjusting only for measured confounders using an appropriate method (e.g., IP-weighted PP, 2SLS, 2SRI) should give us an unbiased effect estimate of the vaccine on flu. Hence, this Setting 1 is a fair scenario for both PP and IV-based methods where PP methods that adjust for *L* and the IV-based methods should all be unbiased.

Using the Eqn. (1), the following are considered to simulate the data for settings 1: *f*_*U*_=Uniform(0,1),*λ*_0_=0,*λ*_1_=6,*σ*=2,*p*_*L*2_=logit(−5+3*U*+1.25*L*_1_),*p*_*A*_=logit(*α*_0_+*α*_1_*Z*+*α*_2_*L*_1_+*α*_3_*L*_2_+*α*_4_*U*), and *p*_*Y*_=logit(*θ*_0_+*θ*_1_*A*+*θ*_2_*L*_1_+*θ*_3_*L*_2_+*θ*_4_*U*+*θ*_5_*Z*). Here, *α*_0_ is associated with the nonadherence rate; *α*_1_=0.6 is the coefficient associated with randomization; *α*_2_=0.4 and *α*_3_=0.35 are coefficients associated with the measured covariates; *α*_4_=0. Also, *θ*_0_ is associated with the event rate; *θ*_1_=0 is the null treatment effect (and thus no arrow from *A* to *Y* in DAG 1); *θ*_2_=0; *θ*_3_=0; *θ*_4_ determines the magnitudes of unmeasured confounders, and *θ*_5_=0 indicates the exclusion restriction is satisfied in this setting. Under different choices of *α*_0_, we considered six levels of deviations from adherence in each arm: 10, 20, 40, 60, 80, and 90%. The parameter choices are summarized in Table 3(A). For each of the nonadherence scenarios, we considered two sets of *θ*_4_ values, making a total of 12 scenarios. We set *θ*_4_ = 8 and 0.5 for strong and weak unmeasured confounders, respectively. The full list of parameters considered for all 12 scenarios for setting 1 is shown in Appendix D Table 5.

**Table 3:** The nonadherence rates per arm for different choices of *α*_0_ for simulation settings 1-3 with (*α*_1_,*α*_2_,*α*_3_,*α*_4_)=(0.6,0.4,0.35,0) for setting 1, (0.25,0.02,0.04,0.05) for setting 2, and (0,0.01,0.04,0) for setting 3

Scenario	Arm	A. Setting 1		B. Setting 2		C. Setting 3	
		*α*_0_	Nonadherence	*α*_0_	Nonadherence	*α*_0_	Nonadherence
1	Z = 1	0.72	10	0.55	11	0.86	10
	Z = 0	-4.06	10	0.02	11	0.06	10
2	Z = 1	-0.23	20	0.46	20	0.76	20
	Z = 0	-3.14	20	0.12	21	0.16	20
3	Z = 1	-1.47	40	0.25	41	0.56	41
	Z = 0	-1.92	40	0.32	41	0.36	40
4	Z = 1	-2.52	60	0.05	61	0.36	60
	Z = 0	-0.85	60	0.52	61	0.57	60
5	Z = 1	-3.76	80	-0.15	81	0.16	80
	Z = 0	0.39	80	0.70	80	0.77	80
6	Z = 1	-4.72	90	-0.25	91	0.06	90
	Z = 0	1.35	90	0.80	89	0.86	90

#### Setting 2: exclusion restriction satisfied, unmeasured confounding present

Palmer et al. [19] described the simulation structure for point-treatment settings where the exclusion restriction assumption of the IV assumption is violated. We slightly modified the structure and considered two versions of this simulation: the exclusion restriction of IV assumption is not violated but there exists unmeasured confounding (Fig. 1(C); DAG 2), and the exclusion restriction assumption is violated (Setting 3; described later). Recall our example on estimating the influenza vaccine effect on flu where information on smoking is unmeasured. As shown in DAG 2, smoking status is a barrier to receive the influenza vaccine (*U*→*A*) [45], but it increases the risk of flu (*U*→*Y*) [44]. Given that the study is a randomized trial, the exclusion restriction could be satisfied in this setting, but there could be unmeasured confounding. Unlike setting 1, adjusting only for measured confounders (e.g., physical activity and illness) may not be sufficient to get an unbiased effect estimate of the vaccine on flu using the PP methods [14].

Using the Eqn. (1), the following are considered to simulate the data for settings 2: *f*_*U*_=Bernoulli(0.5),*λ*_0_=3,*λ*_1_=0,*σ*=0.5,*p*_*L*2_=logit(−3.5+0.6*L*_1_),*p*_*A*_=*α*_0_+*α*_1_*Z*+*α*_2_*L*_1_+*α*_3_*L*_2_+*α*_4_*U*, and *p*_*Y*_=*θ*_0_+*θ*_1_*A*+*θ*_2_*L*_1_+*θ*_3_*L*_2_+*θ*_4_*U*+*θ*_5_*Z*. We considered *α*_1_=0.25,*α*_2_=0.02,*α*_3_=0.04, and *α*_4_=0.05. Under different choices of *α*_0_, we considered six levels of nonadherence: 10, 20, 40, 60, 80, and 90%. The parameter choices are summarized in Table 3(B). For each of the six nonadherence scenarios, we considered five scenarios of the treatment effect of interest (*θ*_1_) and two versions of confounding (*θ*_4_), making a total of 60 scenarios. We set the treatment effect of interest as *θ*_1_={−0.2,−0.05,0,0.05,0.2} and confounding as *θ*_4_ = 0.05 and 0.4 respectively for weak and strong confounding. In this setting, the exclusion restriction is satisfied so that *θ*_5_=0. The complete list of parameters considered for all 60 scenarios for setting 2 is shown in Appendix D Table 6.

#### Setting 3: exclusion restriction violated

If the exclusion restriction is met, *Z* affects the outcome *Y* only through the treatment *A* [36]. For example, if *Z* represents randomization in a double-blind randomized controlled trial, *Z*→*A*→*Y* is expected and the exclusion restriction assumption usually met. However, as discussed by Brookhart et al. [37], if the instrument *Z* represents physician’s prescribing preference, there could be a direct effect of *Z* on *Y* because physicians tend to prescribe selective drugs based on their experience about safety and efficacy of drugs. Recall our motivating example of exploring the effect of the seasonal influenza vaccine on flu. Let *Z* be the physician’s prescribing preference on whether an individual is recommended the seasonal influenza vaccine. Based on the experience, the physicians could prescribe the vaccine only to subjects with a higher risk of the flu [46]. Therefore, in addition to the influence of *Z* on *Y* through *A*, *Z* could directly influence *Y*. The data generating mechanism for this setting is shown in Fig. 1(D; DAG 3). Since the exclusion restriction assumption is violated in this setting, the IV methods (e.g., 2SLS and 2SRI) are expected to produce biased estimates no matter whether we adjust for measured confounders (e.g., physical activity and illness) [24].

Using the Eqn. (1), the following are considered to simulate the data for settings 3: *f*_*U*_=Bernoulli(0.5),*λ*_0_=3,*λ*_1_=0.05,*σ*=0.5,*p*_*L*2_=logit(−3.5+0.6*L*_1_+0.1*U*),*p*_*A*_=*α*_0_+*α*_1_*Z*+*α*_2_*L*_1_+*α*_3_*L*_1_+*α*_4_*U*, and *p*_*Y*_=*θ*_0_+*θ*_1_*A*+*θ*_2_*L*_1_+*θ*_3_*L*_2_+*θ*_4_*U*+*θ*_5_*Z*. We considered *α*_1_=0,*α*_2_=0.01,*α*_3_=0.04, and *α*_4_=0. The same as before, there are six levels of nonadherence for different choices of *α*_0_: 10, 20, 40, 60, 80, and 90%. The parameter choices are summarized in Table 3(C). For each nonadherence scenario, we set two versions of *θ*_1_ and two versions of *θ*_5_, making a total of 24 scenarios. We set *θ*_1_ = 0 for the null treatment effect and 0.2 for the non-null effect. *θ*_5_ determines the severity of the exclusion restriction assumption violation. A small *θ*_5_ value indicates a minor violation of the exclusion restriction, and a large value indicates a severe violation. We set these *θ*_5_ values as 0.05 and 0.2 in this study. The complete list of parameters for these 24 scenarios is shown in Appendix D Table 7.

### Simulation

#### Sample size and iterations

To assess the performance of the estimation methods, we generated 2,000 samples (approximately 1,000 per arm) with 1000 iterations for each scenario based on the above DAGs. R version 4.1.0 was used to perform the analysis.

#### Performance metrics

We assessed the performance of the models through several measures, such as bias, standard error (SE), mean squared error (MSE), and 95% confidence interval (CI) coverage probability. This allows us to compare the performance of different methods in terms of accuracy, precision, and coverage. The following definitions are used to define these measures [47]:

$$ \begin{aligned} \text{Bias} = \frac{1}{n_{\text{sim}}}\sum_{i=1}^{n_{\text{sim}}}{{\hat{\beta}}_{i}-\beta}=\hat{\bar{\beta}}-\beta \\ \text{SE} = \sqrt{\frac{1}{n_{\text{sim}}-1} \sum_{i=1}^{n_{\text{sim}}}{({\hat{\beta}}_{i}-\hat{\bar{\beta}}})^{2}} \\ \text{MSE} = \frac{1}{n_{\text{sim}}}\sum_{i=1}^{n_{\text{sim}}}\left({\hat{\beta}}_{i}-\beta\right)^{2} \\ \text{Coverage} = \frac{1}{n_{\text{sim}}}\sum_{i=1}^{n_{\text{sim}}}{1({\hat{\beta}}_{\text{lower},i}\le\beta\le{\hat{\beta}}_{\text{upper},i})}, \end{aligned} $$

where *β* (i.e., RD) is the true treatment effect of interest, *n*_sim_ is number of iterations (1000 in our case), $\hat {\beta }_{i}$ is the estimated RD in the *i*th iteration, and $\hat {\beta }_{\text {lower},i}$ and $\hat {\beta }_{\text {upper},i}$ are the lower and upper 95% bound of the RD in the *i*th iteration, respectively.

## Results

We presented the results for the baseline-adjusted PP, IP-weighted PP, naive 2SLS, both-stages adjusted 2SLS, naive 2SRI, and both-stages adjusted 2SRI methods in the main text, as they are the main focus of the study. The results for all 14 methods described in Table 1 can be found in Appendix C.

### Setting 1: exclusion restriction satisfied and no unmeasured confounding

#### Bias

**Fig. 2:**
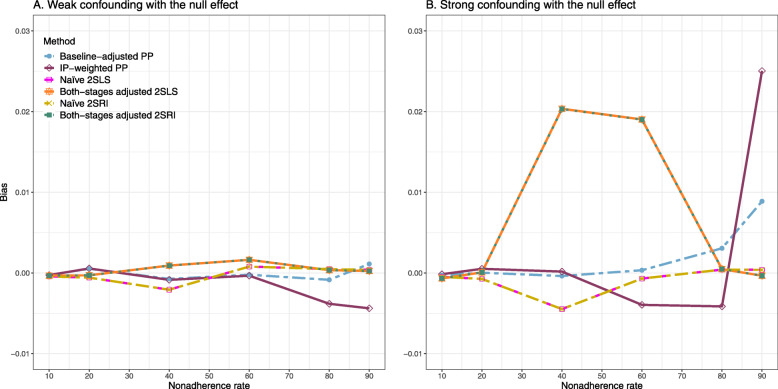
Bias versus the nonadherence rate for simulation setting 1. The 2SLS and 2SRI share the same line, and both-stages adjusted 2SLS and 2SRI share the same line as they produce the same amount of bias. Abbreviations: PP: per-protocol, IP-weighted PP: inverse probability-weighted per-protocol, 2SLS: two-stage least square, 2SRI: two-stage residual inclusion

Figure 2 shows the bias versus an incremental rate of nonadherence using DAG 1 (null treatment effect) for baseline-adjusted PP, IP-weighted PP, naive and both-stages adjusted 2SLS and 2SRI methods. Under the scenario of weak unmeasured confounders, all methods produce small bias. In the presence of strong unmeasured confounders, the 2SLS and 2SRI estimates have nominal biases (range 0 to 0.02). The baseline-adjusted PP and IP-weighted PP estimates are approximately unbiased, but these estimates are biased (the bias is approximately 0.025) beyond 80% of nonadherence. The bias versus nonadherence comparison for all methods considered in this study is shown in Appendix C Fig. 6.

#### SE, MSE, coverage, and bounds of NPCB

The 2SLS and 2SRI methods produce higher SE than PP methods (Appendix C Fig. 7). The baseline-adjusted PP and IP-weighted PP also produce high SE beyond 40% nonadherence. Also, the IP-weighted PP performs the worst beyond 60% nonadherence, producing very high SE (e.g., approximately twice than both-stages adjusted 2SLS and 2SRI methods). The pattern and amount of SE are the same for weak and strong unmeasured confounders scenarios.

Under the null scenario with weak unmeasured confounders, all methods produce small MSE and some share approximately the same line (Appendix C Fig. 8). The naive and both-stages adjusted 2SLS and 2SRI methods have slightly higher MSE compared to the baseline-adjusted PP and IP-weighted PP when the nonadherence rate is ≤60*%*. For example, MSEs are close to zero for the baseline-adjusted PP and IP-weighted PP methods, but about 0.015 for naive and both-stages adjusted 2SLS and 2SRI methods. On the other hand, the baseline-adjusted PP and IP-weighted PP have more than twice MSE than naive and both-stages adjusted 2SLS and 2SRI methods beyond 60% nonadherence. Also, the both-stages adjusted 2SLS and 2SRI produce comparatively smaller MSE than the naive 2SLS and 2SRI methods.

The IP-weighted PP produces nominal 95% coverage for less than 80% nonadherence, but this method produces low coverage for ≥80*%* nonadherence (Appendix C Fig. 9). On the other hand, the naive and both-stages adjusted 2SLS and 2SRI produce nominal 95% coverage regardless of weak or strong unmeasured confounders.

The bounds of the NPCB method using DAG 1 are shown in Appendix C Fig. 10. The NPCB method produces very wide bounds regardless of weak or strong unmeasured confounders. In contrast, the width of bounds is small for 10% and 90% nonadherence, and high for 20% to 80% nonadherence.

### Setting 2: exclusion restriction satisfied, unmeasured confounding present

#### Varying the effect of nonadherence

We presented bias, SE, MSE, and 95% coverage probability for different nonadherence rates. We presented the results for a null (RD = 0) and non-null (RD = 0.2) treatment effect scenarios.

##### Bias

**Fig. 3:**
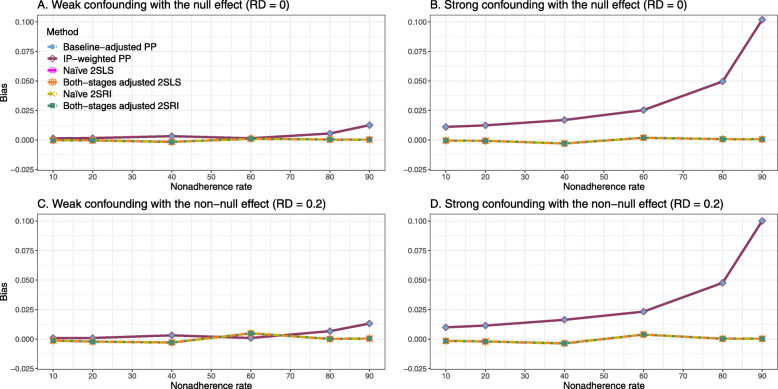
Bias versus the nonadherence rate for the null and non-null effect for simulation setting 2. The naïve and both-stages adjusted 2SLS and 2SRI share the same line as they produce the same amount of bias. The baseline-adjusted PP and IP-weighted PP also share the same line. Abbreviations: RD: risk difference, PP: per-protocol, IP-weighted PP: inverse probability-weighted per-protocol, 2SLS: two-stage least square, 2SRI: two-stage residual inclusion

Under the scenario of weak unmeasured confounding, all methods produce small bias (Fig. 3). In the presence of strong unmeasured confounding, the baseline-adjusted PP and IP-weighted PP produce biased estimates. The amount of bias (range from 0.02 to 0.10) is approximately the same for the null and non-null treatment effect scenario. In contrast, the naive 2SLS and 2SRI, and both-stage adjusted 2SLS and 2SRI methods consistently produce unbiased estimates under the null or non-null and weak or strong confounding scenarios. The bias versus nonadherence rate for all methods using DAG 2 is shown in Appendix C Fig. 11.

##### SE, MSE, coverage, and bounds of NPCB

Appendix C Figs. 12 and 13 show the SE and MSE versus nonadherence using DAG 2, respectively. The baseline-adjusted PP and IP-weighted PP produce low coverage for strong confounding scenarios, and these two methods perform poorly beyond 60% nonadherence (Appendix C Fig. 14). On the other hand, the naive and both-stages adjusted 2SLS and 2SRI consistently have nominal coverage under both null or non-null effects and weak or strong unmeasured confounding scenarios. The NPCB method produces a wide bound for both the null and the non-null effect, regardless of the weak or strong confounding (Appendix C Fig. 15). The width of bounds is very wide for 20% to 80% nonadherence.

#### Varying the effect of the treatment

We presented bias, SE, MSE, and 95% coverage probability for different treatment effects. We showed the results for 10% and 40% nonadherence rates.

##### Bias

**Fig. 4:**
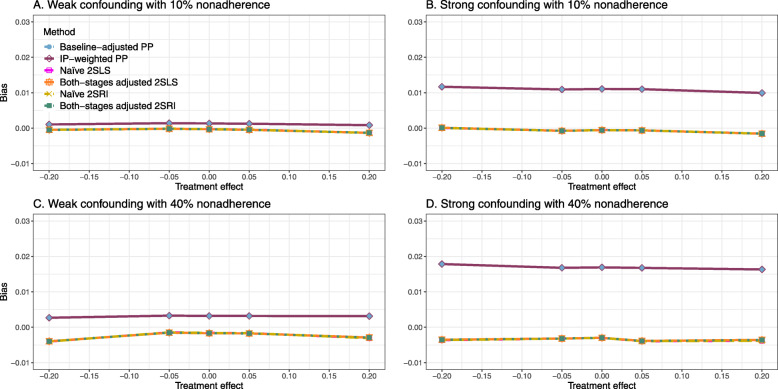
Bias versus treatment effect in risk difference for 10 and 40% nonadherence for simulation setting 2. The naive and both-stages adjusted 2SLS and 2SRI share the same line as they produce the same amount of bias. The baseline-adjusted PP and IP-weighted PP also share the same line. Abbreviations: PP: per-protocol, IP-weighted PP: inverse probability-weighted per-protocol, 2SLS: two-stage least square, 2SRI: two-stage residual inclusion

Figure 4 shows the bias versus treatment effect for 10% and 40% nonadherence using DAG 2. All methods produce approximately an unbiased estimate under the weak confounding scenario with 10% nonadherence. The bias is increased to about 0.015 for baseline-adjusted PP and IP-weighted PP methods under the strong confounding scenario. The bias is more pronounced (about 0.02) for 40% nonadherence. However, the naive and both-stages adjusted 2SLS and 2SRI methods produce approximately unbiased estimates for any treatment effects under weak or strong confounding. The bias versus treatment effect for all methods using DAG 2 is shown in Appendix C Fig. 16.

##### SE, MSE, and coverage

SE and MSE versus treatment effect for 10% and 40% nonadherence using DAG 2 are presented in Appendix C Figs. 17 and 18. The baseline-adjusted PP and IP-weighted PP methods produce small coverage under the strong unmeasured confounding scenario (Fig. 19). On the other hand, the naive and both-stages adjusted 2SLS and 2SRI consistently have nominal coverage regardless of the treatment effect and weak or strong unmeasured confounding.

### Setting 3: exclusion restriction assumption violated

#### Bias

**Fig. 5:**
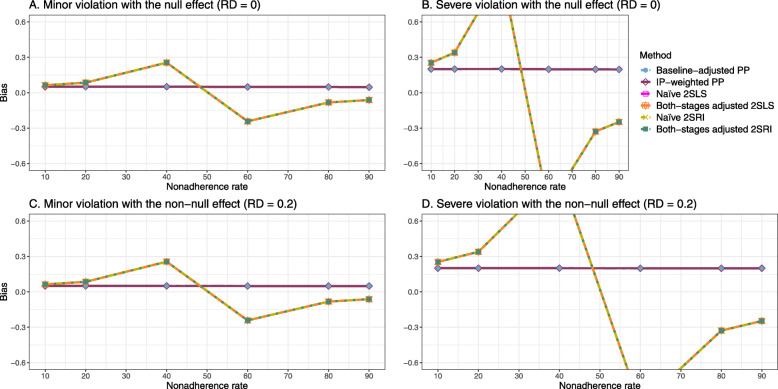
Bias versus the nonadherence rate for the null and non-null effect for simulation setting 3. The 2SLS and 2SRI methods share the same line, and the baseline-adjusted PP and IP-weighted PP share the same line. The bias is out of the bound [-0.6,0.6] for the 2SLS and 2SRI methods for severe violation of the exclusion restriction. Abbreviations: PP: per-protocol, IP-weighted PP: inverse probability-weighted per-protocol, 2SLS: two-stage least square, 2SRI: two-stage residual inclusion

Figure 5 shows the bias versus nonadherence rate using DAG 3. We observed that the 2SLS and 2SRI methods produce high biases when the exclusion restriction assumption is violated. The biases are between -0.3 and 0.3 for a minor violation of the exclusion restriction but beyond the range of [-0.3,0.3] when the violation is severe. The baseline-adjusted PP and IP-weighted PP estimates are also biased for minor or severe violations of the exclusion restriction. However, the bias is slightly smaller (range 0.05 to 0.25) compared to 2SLS and 2SRI methods. The bias versus nonadherence rate for all methods using DAG 3 is shown in Appendix C Fig. 20.

#### SE, MSE, coverage, and bounds of NPCB

The SE and MSE versus nonadherence can be found in Appendix C Figs. 21 and 22. In terms of 95% coverage probability, both PP and IV methods produce low coverage for either minor or severe violations of the exclusion restriction assumption (Appendix C Fig. 23). The NPCB produces wide bounds for all scenarios, regardless of the minor or severe violation of the exclusion restriction assumption (Appendix C Fig. 24).

### Sensitivity analysis

#### Exclusion restriction satisfied, unmeasured confounding present

We produced results for a simplified version of DAG 2 to mimic the possible set of variables as in our case study. The DAG is shown in Appendix C Fig. 25(A) and details are described in Appendix B. The results are shown in Appendix C Figs. 26–29. As expected, the ITT, PP, and AT methods have biased estimates, while the 2SLS and 2SRI methods produce unbiased estimates.

#### Exclusion restriction violated

We produced results when the exclusion restriction assumption is violated, where the setting mimics the possible set of variables as in our case study. The DAG is shown in Appendix C Fig. 25(B) and details are described in Appendix B. The results for bias versus nonadherence are shown in Appendix C Fig. 30. We observed that the 2SLS and 2SRI methods perform worse than even naive methods, no matter whether there is a minor or severe violation of the exclusion restriction assumption.

#### Sensitivity analyses with a smaller sample size

We also simulated data for simulation settings 1-3 by considering 500 samples (approximately 250 per arm). The results are shown in Appendix C Figs. 31–45. The patterns of bias remain the same for all scenarios. As expected, the SEs are higher for the scenarios with 500 samples than those with 2000 samples. Consequently, the MSEs are higher for the scenarios with 500 samples than those with 2000 samples. Also, the IP-weighted PP estimates suffer from non-convergence issues when the nonadherence rate is very high (e.g., 90%). Overall, the conclusions remain the same for the scenarios with a smaller sample size (500) compared to those from a relatively larger sample size (2000). We further attempted generating data by considering 100 samples (approximately 50 per arm), but faced non-convergence issues due to the low sample size, impacting the stability of the estimates; and hence the results are not shown here.

We have added a flow chart showing the present study’s recommendations in Appendix C Fig. 46.

## Case study: vitamin A supplementation on childhood mortality

We used a dataset from a two-arm randomized control trial with 450 villages in northern Sumatra, exploring the effect of vitamin A supplementation on childhood (1-year) mortality [48, 49]. The villages were randomized to either receive vitamin A supplementation or act as a control group for a year. A total of 23,682 children aged 12-71 months from these villages received a large oral dose of vitamin A supplementation (n = 12,094) or no treatment (n = 11,588). Not every child took the assigned vitamin A supplementation in the treatment group, and the nonadherence rate was 20% in the group receiving vitamin A supplements. Vitamin A supplements were not available to children in the control group. This was a randomized trial, and extraneous factors should not influence the instrument *Z* (the randomization variable) [48]. Therefore, there should not be any direct arrow from *Z* to *Y*, or *Z* to *Y* through another pathway other than *A*, meaning that the exclusion restriction assumption for this trial should be reasonably met. Previous studies reported very wide bounds from the same dataset when analyzed by the NPCB method [19, 20]. In the present study, we also assessed the performances of two other IV methods (2SLS and 2SRI) in the same data. Since there was no measured confounder in the dataset, we did not apply baseline covariate-adjusted methods. Instead, we reported the results for the ITT, naive PP, naïve AT, naïve 2SLS, naïve 2SRI, and NPCB methods.

The event rate was 5.1 per 1,000. Table 4 shows the risk difference per 1,000 in the treatment group than the control, standard error, and associated 95% CI. According to the ITT estimate, 2.6 fewer deaths (95% CI: 0.8-4.4) were associated with vitamin A supplementation. However, the ITT estimate do not take into account that a large proportion of children failed to receive vitamin A as prescribed. The PP and AT analyses estimate how efficacious was vitamin A supplementation among those children who actually took the treatment. According to the naive PP, approximately 5.2 less deaths (95% CI: 3.5-6.8) were associated with vitamin A supplementation among those children who actually took the treatment, while it was 6.5 fewer deaths (95% CI: 4.9-8.1) as per naive AT. The 2SLS and 2SRI produce identical results; approximately 3.2 less deaths (95% CI: 1.0-5.5) among the compliers. In contrast, the SE was slightly higher for 2SLS and 2SRI compared to other methods. The bound for the NPCB was reported to be very wide (-5.4 to 194.6) [19, 20].

**Table 4:** Estimated effect of vitamin A supplementation on childhood mortality in a two-arm randomized control trial with 450 villages in northern Sumatra

Method	RD	SE	95% CI
ITT	-2.58	0.93	-4.40, -0.76
Naïve PP	-5.15	0.82	-6.76, -3.53
Naïve AT	-6.47	0.82	-8.08, -4.86
Naïve 2SLS	-3.23	1.16	-5.50, -0.95
Naïve 2SRI	-3.23	1.16	-5.50, -0.96
NPCB ^1^	-	-	-5.39, 194.62

Abbreviations: RD: risk difference per 1,000 in the treatment group than the control; SE: standard error, CI: confidence interval; ITT: intention-to-treat; PP: per-protocol; AT: as-treated; 2SLS: two-stage least square; 2SRI: two-stage residual inclusion; NPCB: nonparametric causal bound;

^1^The interval estimate from the NPCB is the bound for the average causal estimate per 1,000 than a 95% CI.

## Discussion

### Summary of the findings

We used three data-generating mechanisms to study the performance of various statistical methods to deal with nonadherence in pragmatic trial settings with a binary point treatment and a binary outcome. We considered three settings such as (1) exclusion restriction is satisfied and there is no unmeasured confounding; (2) the exclusion restriction assumption is satisfied but there is unmeasured confounding present; (3) the exclusion restriction assumption is violated. No single method is the best in all situations. For setting 1, it is expected that baseline adjusted and IP-weighted PP, and 2SLS and 2SRI estimates are unbiased. We observed that the naive and both-stages adjusted 2SLS and 2SRI methods perform very well in terms of bias and coverage for any nonadherence rate. The baseline-adjusted PP and IP-weighted PP methods outperform the 2SLS and 2SRI methods for below a certain nonadherence rate but show very high bias beyond that point. For our simulation settings, 80% was that cut-point. As expected, the baseline-adjusted PP and IP-weighted PP also have high SE beyond that point. For setting 2, the baseline-adjusted PP and IP-weighted PP perform well in terms of bias, SE, and coverage compared to the 2SLS and 2SRI methods when there is weak unmeasured confounding. However, these PP methods perform poorly under the strong unmeasured confounding. Only naive 2SLS and 2SRI methods and both-stages adjusted 2SLS and 2SRI methods produce approximately unbiased estimates irrespective of weak or strong unmeasured confounding. As expected, these IV methods have high SEs in almost all scenarios. For setting 3, all methods (PP and IV) perform poorly in terms of bias, SE, MSE, and coverage. As expected, the 2SLS and 2SRI methods perform poorly due to the exclusion restriction assumption violation. On the other hand, the baseline-adjusted PP and IP-weighted PP methods also suffer in terms of bias and coverage, but for a different reason: the instrument acts as an unadjusted confounder so that there exists an open backdoor path between the treatment variable and the outcome. For all situations, the first stage adjusted 2SLS and 2SRI have high biases and small coverage probabilities. The NPCB method produces very wide bounds in almost all scenarios, including not capturing the true value in the presence of a severe violation of the exclusion restriction assumption.

### Context in the literature

It is expected from the theory that the ITT, naive PP, and naive AT methods usually produce biased estimates when there is nonadherence [14, 50–52]. The NICE guideline also recommended avoiding these methods in the presence of treatment nonadherence [53].

The adjusted PP methods offer consistently excellent performance under the scenarios without unmeasured confounding [14]. However, it is expected from the literature that the baseline adjusted PP and IP-weighted PP methods produce biased estimates when there is unmeasured confounding [14]. Notably, the presence of unmeasured confounding means the violation of the exchangeability assumption [54]. Using the second definition of the PP methods (i.e., excluding those patients entirely from the analysis), we observed that the adjusted PP methods, such as baseline adjusted PP and IP-weighted PP, have high biases in the presence of strong unmeasured confounding. Therefore, we should not use these methods when it is believed that there is strong unmeasured confounding. On the other hand, the IV-based methods can be used to get the unbiased treatment effect estimate even if some of the necessary confounders are unmeasured [15, 55]. However, the IV methods typically produce a higher SE, which is in-line with the theory [14]. Since the first-stage of the 2SLS and 2SRI is used to predict the treatment, researchers may include covariates only in the first-stage but not in the second stage [28, 29]. Our study showed that adding the covariate only in the first stage of the model but not in the second stage leads to bias estimates. Also, covariates adjustment in IV methods could reduce the SE, which is consistent with the theory [14]. Therefore, we recommend against using first-stage adjusted 2SLS and 2SRI methods, and prefer both-stages adjusted 2SLS or 2SRI methods.

The newer IV-based method, the NPCB, is used by many researchers with the understanding that it can pro- duce a more precise causal bound [20]. The causal bound is a different metric compared to a 95% confidence interval, and hence coverage cannot be calculated directly. Our simulations, however, showed that this method included the true parameter 100% of the time in almost all scenarios, except for a severe violation of the exclusion restriction assumption. Although high SE in 2SLS and 2SRI methods motivate using the NPCB method, this method does not adjust for confounders directly and often produces wide bounds [18–20].

It is also expected from the theory that the IV-methods perform poorly when the key assumption of an IV, such as the exclusion restriction, is violated [24]. The exclusion restriction is untestable using the observed data. Usually, the randomization variable in a trial is an ideal instrument. But a weak instrument can lead to biased and imprecise estimates [56, 57]. Therefore, the 2SLS and 2SRI methods should only be used when this IV assumption is plausible. Subject-matter knowledge should be applied to rule out the possibility of violation of the IV assumption. Besides, the no unmeasured confounding assumption is also untestable from the observed data [58]. As expected, the baseline-adjusted PP and IP-weighted PP perform poorly and producing high bias when there is unmeasured confounding [14]. However, these PP methods outperform the 2SLS and 2SRI methods in terms of bias, MSE, and coverage if there is no or weak unmeasured confounding. Therefore, we suggest collecting information on covariates, when possible, that could influence the treatment nonadherence to rule out the possibility of having strong unmeasured confounding. Based on empirical observations, previous studies proposed using the IV-methods in the presence of unmeasured confounding, while IP-weighted PP can be used to address nonadherence with no unmeasured confounding [4, 59]. In the current work, we applied both of these types of methods under a series of data generating mechanisms and compared the results. We additionally suggest using the both-stages adjusted 2SLS or 2SRI, but not the baseline-adjusted PP or IP-weighted PP method when nonadherence is very high (e.g., ≥80*%* in our case). The IV-based methods can also be used as complementary analysis because the assumptions of these IV methods are different from the non-IV-based methods. If the primary and the complementary analyses result in a similar conclusion, we can have more confidence in the overall conclusion.

### Case study

Using the ITT analysis, 2.6 fewer deaths per 1,000 were associated with vitamin A supplementation was previously reported in the literature [20]. The naive PP and naive AT found 5.2 and 6.5 fewer deaths among those children who actually took the treatment than the control group, respectively. Both naive 2SLS and 2SRI found fewer deaths (RD 3.2) among the compliers. The high estimate by the naive AT is not surprising as this method mostly gives a biased treatment effect when we have treatment nonadherence. The PP estimate is relatively different from the results from 2SLS and 2SRI, might be indicative of the presence of unmeasured confounding. Our second simulation setting shows that the PP methods have a high bias when there is strong unmeasured confounding, but the 2SLS and 2SRI methods still perform very well. Consistent with the literature [19, 20], NPCB method produces a very wide bound, which might be due to the high nonadherence rate, as we observed in our simulation.

### Strengths

The present study has several strengths. We considered three comprehensive simulation settings to explore the performance of different methods to deal with treatment nonadherence. We considered both the null and non-null treatment effect scenarios and low to high nonadherence (e.g., 10% to 90%), which enabled us to compare the methods rigorously. For more than 90% nonadherence, however, most of the algorithms do not converge or produce a very high effect estimate. Moreover, we considered an approximately equal nonadherence rate per arm so that the result has not influenced by the unequal nonadherence rate. We also considered a collapsible effect measure to estimate the treatment effect to ensure that the marginal and conditional treatment effects for any level of the measured confounders are equal and comparable [39]. Utility of our simulation study is that this pedagogic work confirmed theoretical results and helped make the theoretical ideas more accessible to the practitioners and applied researchers. Additionally, we observed similar findings from sensitivity analyses for unmeasured confounding, violation of the exclusion restriction assumption, and small sample size, meaning that our study results are robust.

### Limitations and future direction

Despite its strengths, the study has a few limitations. First, to clearly identify the effect of each factor, we started from a simplistic scenario, but this is easily extendable. Future studies can consider more complex DAGs and add more confounders with both linear and non-linear forms. Second, we did not consider any loss to follow-up, while the differential loss to follow-up can bias the results [5]. The oversimplified point treatment settings and no loss to follow-up also limit guiding about decisions about bias-variance trade-off. Third, our simulation settings have equal nonadherence rates per arm versus one-sided differential nonadherence in our case study. The pattern of bias, SE, MSE, and coverage of all methods under equal versus differential nonadherence are expected to be in the same direction. Our team is exploring this issue in more detail to verify whether differential nonadherence could lead to different conclusions. Fourth, the results in this paper may not be generalizable to other types of outcomes. Some previous studies compared different methods to address treatment nonadherence when the outcome is continuous [60, 61] and time-to-event [26, 62–64]. However, these studies mostly focused on comparing either IV or PP methods with the naive methods, or considered different data structures or treatment strategies (e.g., sustained treatment strategies in longitudinal studies) [26, 64]. Future studies should explore the performance of both IV and PP methods in the point-treatment setting for continuous and time-to-event outcomes, multiple treatment arms, and when effect modifiers are present. In addition to the 2SLS, 2SRI, and NPCB methods, some other IV methods can be used in the same setting to deal with treatment nonadherence. For example, the two-stage predictor substitution (2SPS) can handle non-linearity in the first stage of the 2SLS model and the three-stage least squares method (3SLS) methods can correct the correlation between error terms in the first and second stages of the 2SLS models [65, 66]. The IV-based structural mean model (SMM) is another method that can address treatment nonadherence [67]. This semi-parametric method uses g-estimation for identification and estimation of the treatment effect after addressing the nonadherence issue. Future studies can explore the performance of these IV methods in contrast to the other IV methods and non-IV methods when dealing with the treatment nonadherence in pragmatic trials.

It is also worth mentioning that the 2SRI estimates can be biased even without violating the exclusion restriction assumption [65], and the level of bias may increase as the severity of confounding increases. Cai et al. [65] reported analytic estimates of such bias in terms of log-odds ratios for a variety of situations. In our study, we observed minimal or no increase in bias as the confounding severity increases. This observation might be due to the fact that we were using RD as effect measure, and the impact was less noticeable compared to that when effect measure was odds ratio. Further studies could assess the bias in the 2SRI method when the effect measure is collapsible.

We require the positivity assumption, i.e., non-zero probability of being exposed or unexposed at every combination of the values of the observed confounders [68]. For randomized trials (e.g., as our case study), that probability of being exposed or unexposed is usually known, and so is belonging to one particular confounder category [68]. However, theoretical violations of positivity may arise when patients with certain characteristics may be ineligible to receive a treatment (e.g., absolute contraindication for a given treatment) [69]. If this is the case, the weights from the IP-weighted PP methods can be infinite or very large, and the estimates can be biased and unstable [69]. The high nonadherence rate or small sample size may lead to near positivity violations, which is also responsible for large IP weights. Therefore, even when there is no unmeasured confounding but high nonadherence rates, the biased and large SE in our simulation results could be due to the near positivity violations. Methods such as truncation and overlap weights have been proposed in the literature to deal with the large IP weight problem [70, 71]. On the other hand, a modified causal estimator has been proposed to deal with the positivity issue in the IV estimation [72, 73].

When the treatment is sustained but time-varying, the same methods that we considered in this study are not generally adequate, and methods have been extended to address those scenarios [26, 27]. Naïve methods such as ITT, naïve PP, or naïve AT ignore time-varying confounding and can produce biased estimates [9]. There exists some new literature exploring nonadherence in a sustained treatment strategy when longitudinal post-baseline prognostic variable measures are available, which is beyond the scope of our point-treatment study.

## Conclusion

Besides the research question, the choice of the methods should come from researcher’s understanding of the underlying data generating mechanism and available data (e.g., extent of unmeasured confounding and a potential violation of exclusion restriction). An additional consideration includes the nonadherence rate (e.g., high vs. low). Under nearly an ideal situation where there are unmeasured confounders but adjusting for measured confounders can give an unbiased effect estimate, the naive and both-stages adjusted but not first-stage adjusted 2SLS and 2SRI methods perform very well in terms of bias and coverage for any nonadherence rate. Although the naive and both-stages adjusted 2SLS and 2SRI methods give almost identical bias, both-stages adjusted 2SLS and 2SRI methods improve the precision and reduce overall MSE. In the same setting, the baseline-adjusted PP and IP-weighted PP outperform these 2SLS and 2SRI methods in terms of bias, SE, and MSE for <80% nonadherence, but these PP methods show very high bias and MSE for ≥80*%* nonadherence rate. If there is no unmeasured confounding, the baseline-adjusted PP and IP-weighted PP consistently outperform the 2SLS and 2SRI methods in terms of bias, SE, MSE, and coverage. However, these two PP methods perform poorly when some necessary confounders are unmeasured and measured confounders cannot sufficiently block the backdoor paths between the treatment and the outcome. On the other hand, the 2SLS and 2SRI produce high biases and low coverage probabilities when there exists violation of the exclusion restriction assumption. The baseline-adjusted PP and IP-weighted PP can also have biased estimates when the exclusion restriction is violated. However, the baseline-adjusted PP and IP-weighted PP can produce unbiased estimates if all open backdoor paths between the treatment variable and the outcome can be blocked so that the association between the instrument and outcome is nullified. Therefore, when possible, we recommend collecting information on necessary covariates that predict adherence and addressing them appropriately in the analyses. Collecting information on those covariates or augmentation of external data sources from electronic health records could reduce the impact of having strong unmeasured confounding. Since assumptions of PP methods and IV methods are different and often untestable, we suggest analyzing the data using both PP methods (baseline-adjusted PP or IP-weighted PP) and IV-methods (both-stages adjusted 2SLS or 2SRI) and reporting both results. Analysts are more likely to come up with a robust conclusion of the real-world effect of a treatment if they have similar findings from different analyses requiring different assumptions.

## Appendix A

This section describes the estimation methods considered in this study and the assumptions of these estimation methods.

### Description of the estimation methods

Let *Z* is the two-arm randomization variable, *A* is the binary treatment variable, *L*=(*L*_1_,*L*_2_) is a vector of measured confounders, and *Y* is the binary outcome. The risk difference (RD) is our target parameter of interest. The binomial model with an identity link function can be used to estimate RD. Since the binomial model fitting frequently shows convergence issues with adjusting for covariates in the model, the Poisson or Gaussian regression with an identity link function and robust sandwich standard error can be used as an alternative [40]. In the present study, we considered intention-to-treat (ITT), naive per-protocol (PP), naive as-treated (AT), baseline adjusted ITT, baseline adjusted PP, IP-weighted PP, two-stage least square (2SLS), two-stage residual inclusion (2SRI), and non-parametric causal bound (NPCB) methods to estimate RD and associated parameters for a two-arm pragmatic trial. The description of calculating RD using these methods is given below.

#### ITT

The ITT models *Z* on *Y* without adjustment for *L*. The model can be written as

$$\Pr(Y = 1) = \beta_{0} + \beta_{\text{ITT}} Z. $$

Then $\hat {\beta }_{\text {ITT}}$ is the maximum likelihood estimate (MLE) of *β*_ITT_ and is the estimated RD.

#### Naive PP

The naive PP models *Z* on *Y* among those subjects who receive the treatment according to the protocol (*Z*=*A*) but without adjustment for *L*. The model can be written as:

$$\Pr(Y = 1) = \beta_{0} + \beta_{\text{Naive-PP}} Z \quad \text{for Z=A}. $$

Then $\hat {\beta }_{\text {Naive-PP}}$ is the estimated RD.

#### Naive AT

The naive AT models *A* on *Y*, but does not consider whether individuals randomized to the treatment groups. The model cannot adjust for *L* and can be written as:

$$\Pr(Y = 1) = \beta_{0} + \beta_{\text{Naive-AT}} A. $$

Then $\hat {\beta }_{\text {Naive-AT}}$ is the estimated RD.

#### Baseline-adjusted ITT

The baseline-adjusted ITT is the same as ITT but it adjusts for *L*:

$$\Pr(Y = 1) = \beta_{0} + \beta_{\text{B-ITT}} Z + \beta_{2}L_{1} + \beta_{3}L_{2}. $$

Then $\hat {\beta }_{\text {B-ITT}}$ is the estimated RD.

#### Baseline-adjusted PP

The baseline-adjusted PP is the same as naive PP but it adjusts for *L*:

$$\Pr(Y = 1) = \beta_{0} + \beta_{\text{B-PP}} Z + \beta_{2}L_{1} + \beta_{3}L_{2} \quad \text{for Z=A}. $$

Then $\hat {\beta }_{\text {B-PP}}$ is the estimated RD.

#### IP-weighted PP

The method creates inverse probability adherence weights to generate a pseudo population to estimate the treatment effect by removing the effect of nonadherence [25]. We used a logistic regression to estimate the adherence probabilities among those subjects who receive the treatment according to the protocol (*Z*=*A*) as follows

$$\text{logit}(\Pr(A = 1)) = \gamma_{0} + \gamma_{1}L_{1} + \gamma_{2}L_{2}. $$

The predicted probability from the above model is the probability of adherence *P*_*A*_. We calculated the stabilized weights to prevent from extreme weights [7, 30]. The stabilized inverse probability of adherence weights can be calculated as

$$\mathrm{W}_{\text{stabilized}} = \Pr(A=1)\frac{A}{P_{A}} + \Pr(A=0)\frac{1-A}{1-P_{A}}. $$

To estimate the RD, the weighted outcome model is used, which can be written as

$$\Pr(Y = 1) = \beta_{0} + \beta_{\text{IPW-PP}} Z \quad \text{with weight = }\mathrm{W}_{\text{stabilized}}. $$

Then $\hat {\beta }_{\text {IPW-PP}}$ is the estimated RD.

#### Naïve 2SLS

There are two stages in the 2SLS method. The instrument (*Z*) is modelled to *A* in the first stage, and then the predicted treatment is modelled to *Y* in the second stage [31]. There is no adjustment for *L* in either stage of the model. We used a logistic regression in the first stage of the model, which can be written as

$$\text{logit}(\Pr(A=1))=\gamma_{0}+\gamma_{1} Z. $$

Then the second stage model can be written as follows

$$\Pr(Y = 1) = \beta_{0} + \beta_{\text{Naive-2SLS}}\widehat{A}, $$

where $\hat {A}$ is the predicted treatment from the first stage, and then $\hat {\beta }_{\text {Naive-2SLS}}$ is the estimated RD.

#### First-stage adjusted 2SLS

The first-stage adjusted 2SLS is the same as naive 2SLS except it adjusts for *L* in the first stage of the model [28, 29]. The same as naive 2SLS, we used a logistic regression in the first stage of the model

$$\text{logit}(\Pr(A=1)) = \gamma_{0}+\gamma_{1} Z + \gamma_{2}L_{1} + \gamma_{3}L_{2}. $$

Then the second stage model can be written as follows

$$\Pr(Y = 1) = \beta_{0} + \beta_{\text{1Stage-2SLS}}\widehat{A}, $$

where $\hat {A}$ is the predicted probabilities for *A* from the first stage, and then $\hat {\beta }_{\text {1Stage-2SLS}}$ is the estimated RD.

#### Both-stages adjusted 2SLS

The both-stages adjusted 2SLS is the same as naive 2SLS except it adjusts for *L* in both stages of the model. The same as naive and first-stage adjusted 2SLS, we used a logistic regression in the first stage of the model

$$\text{logit}(\Pr(A=1)) = \gamma_{0}+\gamma_{1} Z + \gamma_{2}L_{1} + \gamma_{3}L_{2}. $$

Then the second stage model can be written as follows

$$\Pr(Y = 1) = \beta_{0} + \beta_{\text{BStage-2SLS}}\widehat{A} + \beta_{2}L_{1} + \beta_{3}L_{2}, $$

where $\hat {A}$ is the predicted probabilities for *A* from the first stage, and then $\hat {\beta }_{\text {BStage-2SLS}}$ is the estimated RD.

#### Naïve 2SRI

Similar to the 2SLS, there are two stages in the 2SRI method. But the instrument (*Z*) is modelled to *A* in the first stage, and then the residuals from the first stage and *A* are modelled to *Y* in the second stage [22]. There was no adjustment for *L* in either stage of the model. We used a logistic regression in the first stage of the model, which can be written as

$$\text{logit}(\Pr(A=1)) = \gamma_{0}+\gamma_{1} Z. $$

The residuals from the first stage can be extracted as follows:

$$r = A - \widehat{A}, $$

where $\hat {A}$ is the predicted probability for *A* from the first stage. Then the second stage model can be written as follows

$$\Pr(Y = 1) = \beta_{0} + \beta_{\text{Naive-2SRI}}A+\beta_{2} r, $$

and then $\hat {\beta }_{\text {Naive-2SRI}}$ is the estimated RD.

#### First-stage adjusted 2SRI

The first-stage adjusted 2SRI is the same as naive 2SRI except it adjusts for *L* in the first stage of the model [29]. The same as naive 2SRI, we used a logistic regression in the first stage of the model

$$\text{logit}(\Pr(A=1)) = \gamma_{0}+\gamma_{1} Z + \gamma_{2}L_{1} + \gamma_{3}L_{2}. $$

The same as before, the residuals from the first stage can be extracted as follows:

$$r = A - \widehat{A}, $$

where $\hat {A}$ is the predicted probability for *A* from the first stage. Then the second stage model can be written as follows

$$\Pr(Y = 1) = \beta_{0} + \beta_{\text{1Stage-2SRI}}A+\beta_{2} r, $$

and then $\hat {\beta }_{\text {1Stage-2SRI}}$ is the estimated RD.

#### Both-stages adjusted 2SRI

The both-stages adjusted 2SRI is the same as naive 2SRI except it adjusts for *L* in both stages of the model [22]. The same as naive and first-stage adjusted 2SRI, we used a logistic regression in the first stage of the model

$$\text{logit}(\Pr(A=1)) = \gamma_{0}+\gamma_{1} Z + \gamma_{2}L_{1} + \gamma_{3}L_{2}. $$

Then the second stage model can be written as follows

$$\Pr(Y = 1) = \beta_{0} + \beta_{\text{1Stage-2SRI}}A+\beta_{2} r + \beta_{2}L_{1} + \beta_{3}L_{2}, $$

where $r = A - \widehat {A}$ is the residuals from the first stage with $\hat {A}$ is the predicted probability for *A* from the first stage. Then $\hat {\beta }_{\text {1Stage-2SRI}}$ is the estimated RD.

#### NPCB

This nonparametric method estimates the bounds for the effect of interest rather than a point estimate and is restricted to a binary outcome (*Y*) with a binary or trinary instrument [18, 19]. This method uses a constrained probability statement to provide bounds on the estimated treatment effect. But these bounds are the range of the true causal effect of interest, not the confidence interval [18, 19]. The bounds for the RD can be written as

$$\begin{array}{*{20}l} &\max \left \{ \begin{array}{c} p_{00.0} + p_{11.1} - 1 \\ p_{00.1} + p_{11.1} - 1 \\ p_{11.0} + p_{00.1} - 1 \\ p_{00.0} + p_{11.0} - 1 \\ 2p_{00.0} + p_{11.0} + p_{11.0} + p_{11.1} - 2 \\ p_{00.0} + 2_{p11.0} + p_{00.1} + p_{01.1} - 2 \\ p_{10.0} + p_{11.0} + 2_{p00.1} + p_{11.1} - 2 \\ p_{00.0} + p_{01.0} + p_{00.1} + 2p_{11.1} - 2 \end{array} \right \} \le \text{RD}\\ &\le \min \left \{ \begin{array}{c} 1 - p_{10.0} - p_{01.1} \\ 1 - p_{01.0} - p_{10.1} \\ 1 - p_{01.0} - p_{10.0} \\ 1 - p_{01.1} - p_{10.1} \\ 2 - 2p_{01.1} - p_{10.0} - p_{10.1} - p_{11.1} \\ 2 - p_{01.0} - 2_{p10.0} - p_{00.1} - p_{01.1} \\ 2 - p_{10.0} - p_{11.0} - 2p_{01.1} - p_{10.1} \\ 2 - p_{00.0} - p_{01.0} - p_{01.1} - 2_{p10.1} \end{array} \right \}, \end{array} $$

where *p*_*ya*.*z*_= Pr(*Y*=*y*, *A*=*a*|*Z*=*z*) with 0≤*p*_*ya*.*z*_≤1 and $\sum _{y,a}p_{ya.z}=1$ [19].

### Assumptions of the estimation methods

To estimate a causal effect, all models described in the Methods section assume consistency (i.e., the potential outcome under the observed treatment is the observed outcome), no interference (i.e., potential outcome for a subject does not depend on the treatment status of another subject), exchangeability (i.e., no unmeasured confounding), positivity (i.e., probability of receiving either treatment is greater than zero), and well-defined interventions (e.g., taking 5mg aspirin) [74, 75]. All parametric methods (all methods except the NPCB) further assume correct model specification [76]. The naive methods assume a random pattern of nonadherence [4]. The IV-based methods assume the IV is associated with the treatment (relevance assumption), there are no defiers (monotonicity assumption), and the IV affects the outcome only through its effect on treatment [77]. The third assumption is also known as the exclusion restriction. Additionally, the 2SRI assumes the linearity of residuals, i.e., residuals are linearly associated with the outcome of interest [10, 77]. Furthermore, all methods assume missing at random and no measurement error [78].

## Appendix B

This section is for the results of sensitivity analyses. The DAGs are shown in Appendix C Fig. 25. Compared to the main text DAG, there is no measured confounder for these appendix DAGs. We consider these simplified scenarios to replicate the scenario for our case study.

### Sensitivity analysis: exclusion restriction satisfied, unmeasured confounding present

#### Setup

This sensitivity analysis was done for a simplified version of DAG 2 in the main text, where the exclusion restriction is satisfied but unmeasured confounding is present. The DAG is shown in Appendix C Fig. 25(A). For this scenario, we simulated data from the following algorithms, where *A* is the treatment, *Y* is the outcome of interest, *Z* is the randomization, and *U* is unmeasured confounding.

2 $$ \begin{aligned} Z \sim \text{Bernoulli}(0.5) \\ U \sim \text{Bernoulli}(0.5) \\ A \sim \text{Bernoulli}\left(p_{A}\right)\ \text{with} \ p_{A} = \alpha_{0} + \alpha_{1} Z + \alpha_{4} U \\ Y \sim \text{Bernoulli}\left(p_{Y}\right)\ \text{with} \ p_{Y} = \theta_{0} + \theta_{1} A + \theta_{4} U + \theta_{5} Z. \end{aligned}  $$

In Eqn. (2), *α*_0_ is associated with the nonadherence rate, *α*_1_=0.1 is the coefficient associated with *Z*, *α*_4_=0.1 is the coefficient associated with *U*; *θ*_0_ is associated with the event rate, *θ*_1_ is the treatment effect of interest, and *θ*_4_ determines the strength of confounding, and *θ*_5_ determines the strength of the direct effect of IV assumption of exclusion restriction violation. Under different choices of *α*_0_, we considered six levels of nonadherence: 10, 20, 40, 60, 80, and 90%.

We set *θ*_0_=0.2, and *θ*_5_=0 (i.e., no violation of exclusion restriction assumption). For each of the six nonadherence scenarios (*α*_0_), we considered five scenarios of the treatment effect of interest (*θ*_1_) and two versions of confounding (*θ*_4_), making a total of 60 scenarios. We set the treatment effect of interest as *θ*_1_={−0.2,−0.05,0,0.05,0.2} and confounding as *θ*_4_ = 0.1 and 0.5 respectively for weak and strong confounding.

#### Results

Since there was no measured confounder (*L*) associated with our data generating mechanism here, there are no baseline adjusted methods as well as first-stage adjusted or both-stages adjusted 2SLS and 2SRI methods. Instead, we reported the results for the ITT, naive PP, naive AT, naive 2SLS, naive 2SRI, and NPCB methods.

Under the null treatment effect scenario with weak confounding, all methods produce small bias (Appendix C Fig. 26). In the presence of strong confounding, the naive PP and AT produce slightly large bias. The amount of bias remains approximately the same for the non-null treatment effect. The ITT method produces a very small bias for the null effect, while it performs the worst when the treatment effect is non-null. In contrast, the 2SLS and 2SRI produce unbiased estimates in all scenarios.

Under the null treatment effect scenario, all methods produce small MSE in the presence of weak confounding, while naive PP shows higher MSE beyond 60% of nonadherence (Appendix C Fig. 27). Under the non-null effect, we observed a similar pattern of MSE except for the ITT. The ITT method produces very high MSE regardless of weak or strong confounding.

Appendix C Fig. 28 shows the 95% coverage probability for different nonadherence rates using Appendix DAG 1. As expected, the ITT method has the highest coverage for the null treatment effect scenario, while it has minimal coverage for the non-null effect. The naive PP and AT methods also produce noticeable small coverage when there is weak confounding, but these methods produce very small coverage under the strong confounding scenarios. On the other hand, naive 2SLS and 2SRI consistently have very high coverage under both null or non-null effects and weak or strong unmeasured confounding scenarios.

The NPCB method produces a wider bound for both the null and the non-null effect, regardless of the weak or strong confounding (Appendix C Fig. 29). However, the width of bounds is only smaller for 10% and 90% nonadherence.

### Sensitivity analysis: violation of the exclusion restriction

#### Setup

In this example, the exclusion restriction is violated (*Z* directly affects *Y*) and there exists an unmeasured confounder *U*. The DAG is shown in Appendix C Fig. 25(B). We used Eqn. (2) to generate the data for Appendix DAG 2. We set *θ*_0_=0.2,*θ*_1_=0.05, two versions of *θ*_4_, and two versions of *θ*_5_ with 10, 20, 40, 60, 80, and 90% nonadherence, making a total of 24 scenarios. We set *θ*_5_ = 0.2 meaning that the exclusion restriction of the IV assumption violation is larger and *θ*_5_ = 0.05 indicating the violation is smaller. The same as Appendix DAG 1, we set *θ*_4_ = 0.1 and 0.5 for weak and strong confounding, respectively.

#### Results

Since there was no measured confounder (*L*) associated with our data generating mechanism here, there are no baseline adjusted methods as well as first-stage adjusted or both-stages adjusted 2SLS and 2SRI methods. Instead, we reported the results for the ITT, naive PP, naive AT, naive 2SLS, naive 2SRI, and NPCB methods.

Appendix C Fig. 30 shows the bias versus nonadherence rate using Appendix DAG 2. Appendix C Fig. 30(A) and (B) show the results when the violation of the exclusion restriction assumption is minor, and Appendix C Fig. 30(C) and (D) show the results when violation is severe. We observed that the 2SLS and 2SRI methods produce a greater bias when the exclusion restriction assumption is violated. The bias is more pronounced when the violation of exclusion restriction assumption is severe.

## Appendix C

This section contains all the supplementary figures considered in this study.

### Supplementary figures for simulation setting 1

**Fig. 6:**
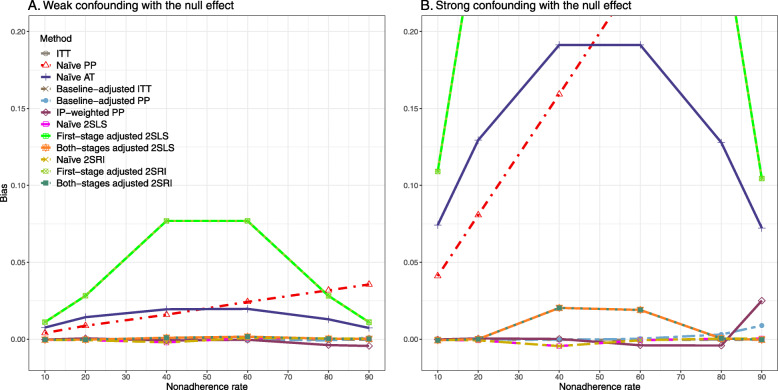
Bias versus the nonadherence rate using DAG 1. The naive 2SLS and 2SRI share the same line, the first stage adjusted 2SLS and 2SRI share the same line, and both-stages adjusted 2SLS and 2SRI share the same line as they produce the same amount of bias. The first stage adjusted 2SLS and 2SRI, and naive PP produce larger than 0.20 bias for strong unmeasured confounders so that the bias is out of the bound [0, 0.20]. Abbreviations: ITT: intention-to-treat, PP: per-protocol, AT: as-treated, IP-weighted PP: inverse probability-weighted per-protocol, 2SLS: two-stage least square, 2SRI: two-stage residual inclusion

**Fig. 7:**
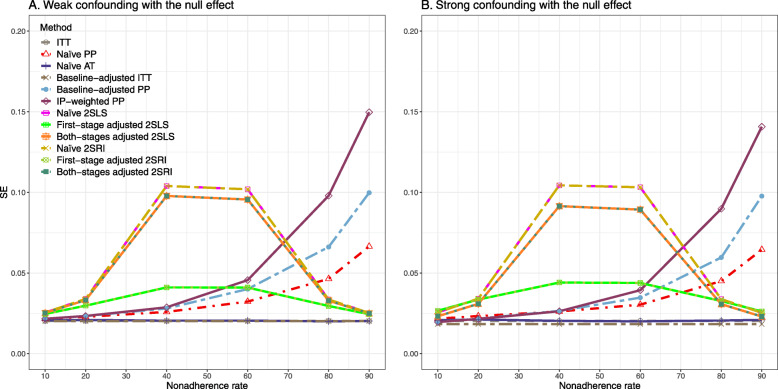
Standard error (SE) versus the nonadherence rate using DAG 1. The naïve 2SLS and 2SRI share the same line, the first stage adjusted 2SLS and 2SRI share the same line, and both-stages adjusted 2SLS and 2SRI share the same line as they produce approximately the same SE. Abbreviations: ITT: intention-to-treat, PP: per-protocol, AT: as-treated, IP-weighted PP: inverse probability-weighted per-protocol, 2SLS: two-stage least square, 2SRI: two-stage residual inclusion

**Fig. 8:**
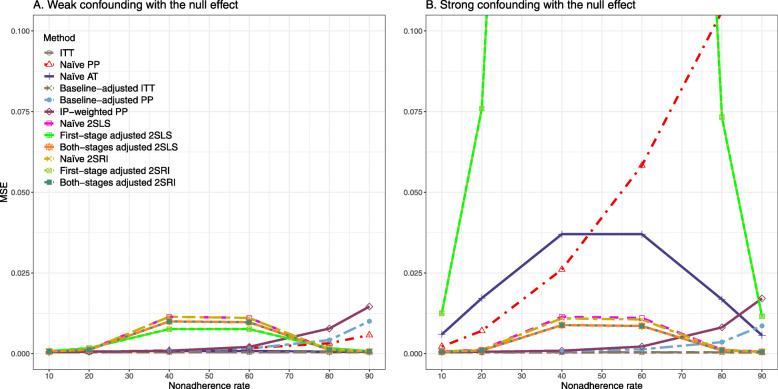
Mean squared error (MSE) versus the nonadherence rate for the null effect using DAG 1. The naïve 2SLS and 2SRI share the same line, the first stage adjusted 2SLS and 2SRI share the same line, and both-stages adjusted 2SLS and 2SRI share the same line as they produce approximately the same MSE. The first stage adjusted 2SLS and 2SRI, and naïve PP produce MSEs larger than 0.10 for strong unmeasured confounders so that the MSE is out of the bound [0, 0.1]. Abbreviations: ITT: intention-to-treat, PP: per-protocol, AT: as-treated, IP-weighted PP: inverse probability-weighted per-protocol, 2SLS: two-stage least square, 2SRI: two-stage residual inclusion

**Fig. 9:**
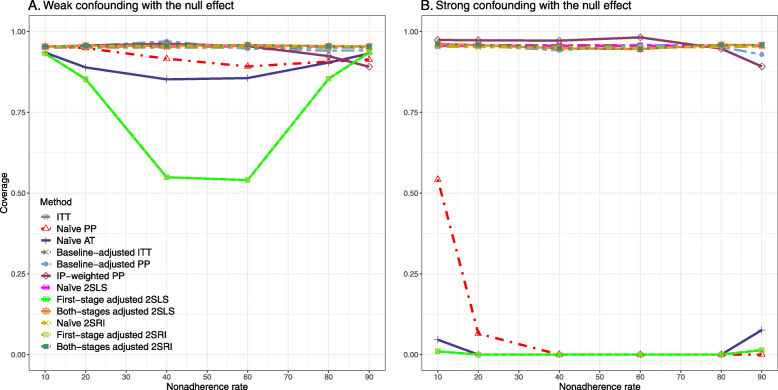
95% coverage probability versus the nonadherence rate using DAG 1. The naïve 2SLS and 2SRI share the same line, the first-stage adjusted 2SLS and 2SRI share the same line, and both-stages adjusted 2SLS and 2SRI share the same line as they produce approximately the same coverage probability. Abbreviations: ITT: intention-to-treat, PP: per-protocol, AT: as-treated, IP-weighted PP: inverse probability-weighted per-protocol, 2SLS: two-stage least square, 2SRI: two-stage residual inclusion

**Fig. 10:**
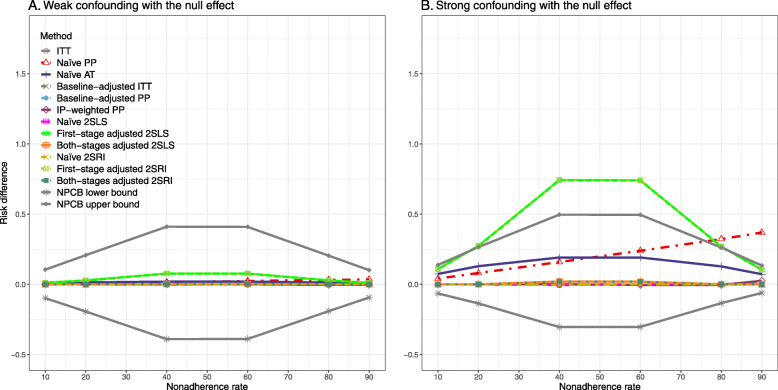
Mean risk difference (RD) versus the nonadherence rate for the null effect using DAG 1. The grey lines are the lower and upper bound of the NPCB method. The naïve 2SLS and 2SRI share the same line, the first stage adjusted 2SLS and 2SRI share the same line, and both-stages adjusted 2SLS and 2SRI share the same line as they produce the same RD estimate. Abbreviations: ITT: intention-to-treat, PP: per-protocol, AT: as-treated, IP-weighted PP: inverse probability-weighted per-protocol, 2SLS: two-stage least square, 2SRI: two-stage residual inclusion, NPCB: nonparametric causal bound

### Supplementary figures for simulation setting 2

**Fig. 11:**
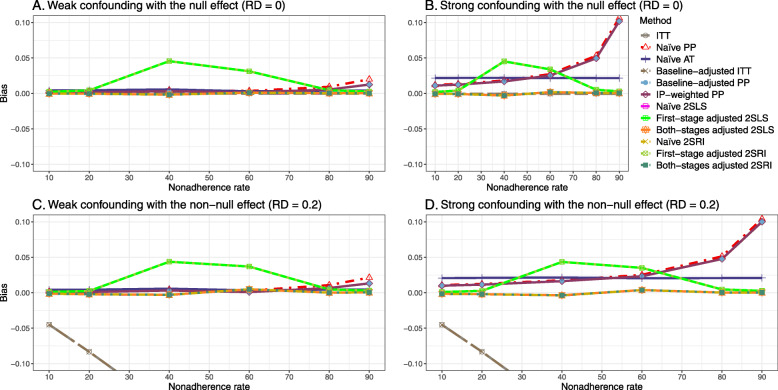
Bias versus the nonadherence rate for the null and non-null effect using DAG 2. The naïve 2SLS and 2SRI share the same line, the first stage adjusted 2SLS and 2SRI share the same line, and both-stages adjusted 2SLS and 2SRI share the same line as they produce the same amount of bias. The bias for the ITT and baseline-adjusted ITT are out of the bound [-0.1,0.1] for the non-null effect. Abbreviations: RD: risk difference, ITT: intention-to-treat, PP: per-protocol, AT: as-treated, IP-weighted PP: inverse probability-weighted per-protocol, 2SLS: two-stage least square, 2SRI: two-stage residual inclusion

**Fig. 12:**
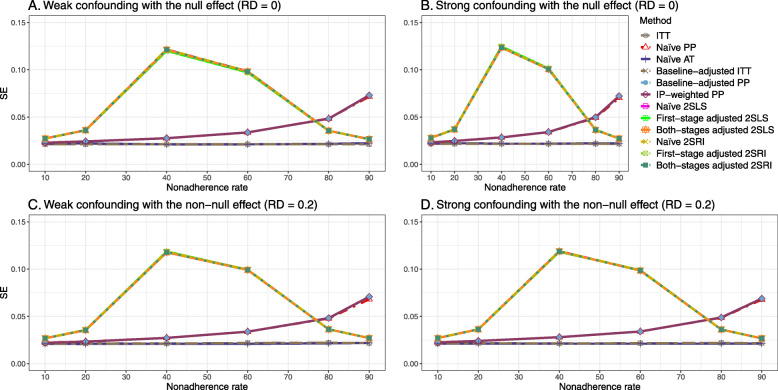
Standard error (SE) versus the nonadherence rate for null and non-null effect using DAG 2. All 2SLS and 2SRI methods share the same line as they produce approximately the same SE. The PP methods also share approximately the same line. Abbreviations: RD: risk difference, ITT: intention-to-treat, PP: per-protocol, AT: as-treated, IP-weighted PP: inverse probability-weighted per-protocol, 2SLS: two-stage least square, 2SRI: two-stage residual inclusion

**Fig. 13:**
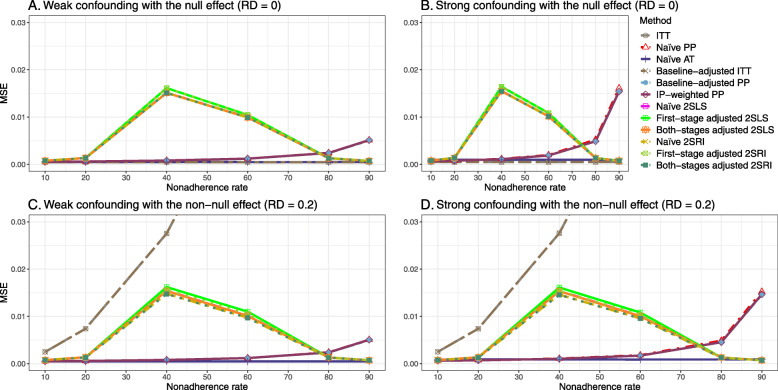
Mean squared error (MSE) versus the nonadherence rate for null and non-null effect using DAG 2. The 2SLS and 2SRI share the same line as they produce approximately the same amount of MSE. The ITT and baseline-adjusted ITT produce MSEs larger than 0.03 for the non-null effect scenario so that the MSE is out of the bound [0, 0.03]. Abbreviations: RD: risk difference, ITT: intention-to-treat, PP: per-protocol, AT: as-treated, IP-weighted PP: inverse probability-weighted per-protocol, 2SLS: two-stage least square, 2SRI: two-stage residual inclusion

**Fig. 14:**
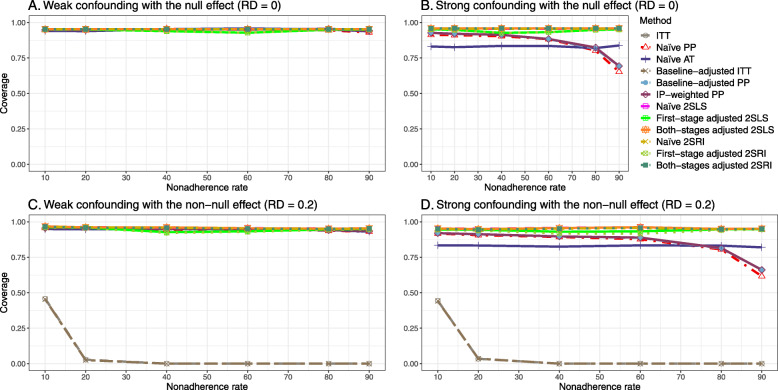
95 percentage coverage probability versus the nonadherence rate for the null and non-null effect using DAG 2. The naive and both-stages adjusted 2SLS and 2SRI share the same line as they produce approximately the same coverage probability. Abbreviations: RD: risk difference, ITT: intention-to-treat, PP: per-protocol, AT: as-treated, IP-weighted PP: inverse probability-weighted per-protocol, 2SLS: two-stage least square, 2SRI: two-stage residual inclusion

**Fig. 15:**
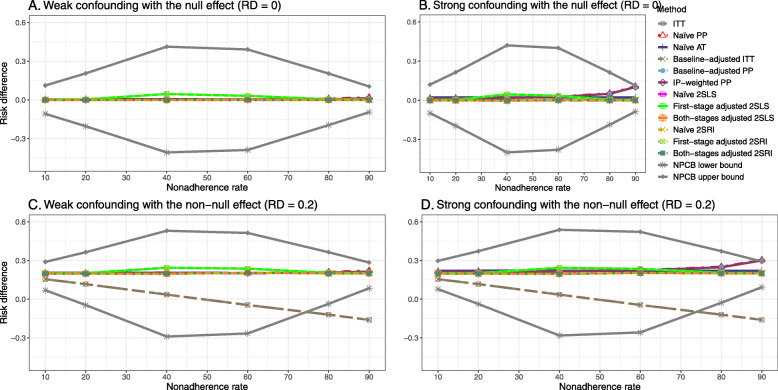
Mean risk difference (RD) versus the nonadherence rate for DAG 2. The grey lines are the lower and upper bound of the NPCB method. The ITT and baseline-adjusted ITT superimposed on each other. Abbreviations: RD: risk difference, ITT: intention-to-treat, PP: per-protocol, AT: as-treated, IP-weighted PP: inverse probability-weighted per-protocol, 2SLS: two-stage least square, 2SRI: two-stage residual inclusion, NPCB: nonparametric causal bound

**Fig. 16:**
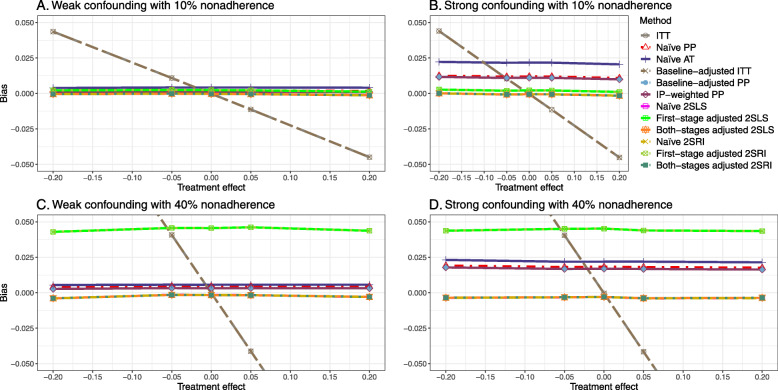
Bias versus treatment effect in risk difference for 10 percent and 40 percent nonadherence using DAG 2. The naive and both-stages adjusted 2SLS and 2SRI share the same line as they produce the same amount of bias. The first-stage adjusted 2SLS and 2SRI methods also share the same line. The ITT and baseline-adjusted ITT produce high bias for 40 percent nonadherence so that the bias is out of the bound [-0.05, 0.05]. Abbreviations: ITT: intention-to-treat, PP: per-protocol, AT: as-treated, IP-weighted PP: inverse probability-weighted per-protocol, 2SLS: two-stage least square, 2SRI: two-stage residual inclusion

**Fig. 17:**
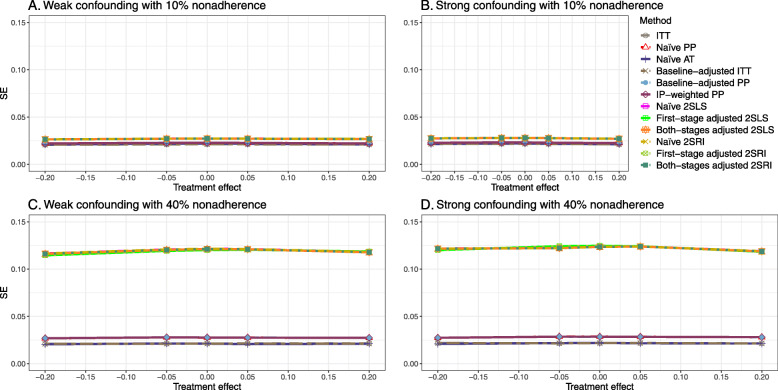
Standard error (SE) versus treatment effect in risk difference for 10 percent and 40 percent nonadherence using DAG 2. All 2SLS and 2SRI methods share the same line as they produce approximately the same SE. The PP methods also superimposed on each other. Abbreviations: ITT: intention-to-treat, PP: per-protocol, AT: as-treated, IP-weighted PP: inverse probability-weighted per-protocol, 2SLS: two-stage least square, 2SRI: two-stage residual inclusion

**Fig. 18:**
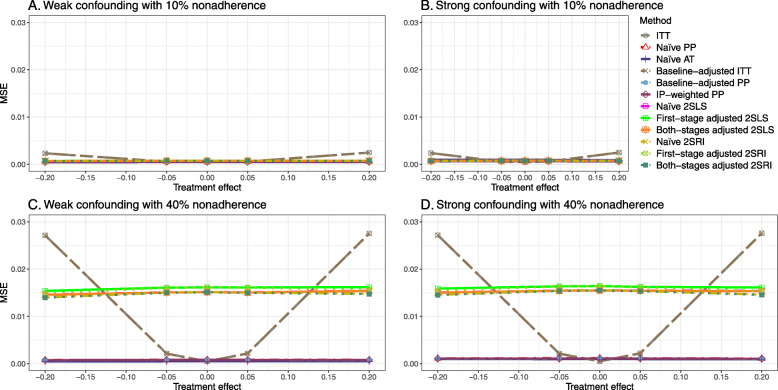
Mean squared error (MSE) versus treatment effect in risk difference for 10 percent and 40 percent nonadherence using DAG 2. Abbreviations: ITT: intention-to-treat, PP: per-protocol, AT: as-treated, IP-weighted PP: inverse probability-weighted per-protocol, 2SLS: two-stage least square, 2SRI: two-stage residual inclusion

**Fig. 19:**
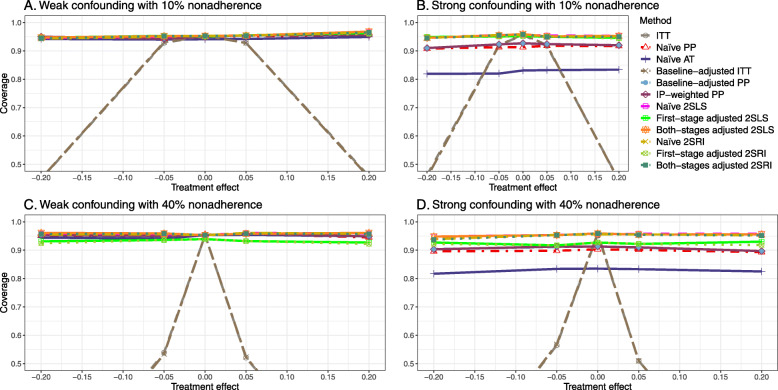
95 percentage coverage probability versus treatment effect in risk difference for 10 percent and 40 percent nonadherence using DAG 2. The coverage probability for the ITT and baseline-adjusted ITT is less than 0.5 for some scenarios and out of the bound [0.5, 1]. Abbreviations: ITT: intention-to-treat, PP: per-protocol, AT: as-treated, IP-weighted PP: inverse probability-weighted per-protocol, 2SLS: two-stage least square, 2SRI: two-stage residual inclusion

### Supplementary figures for simulation setting 3

**Fig. 20:**
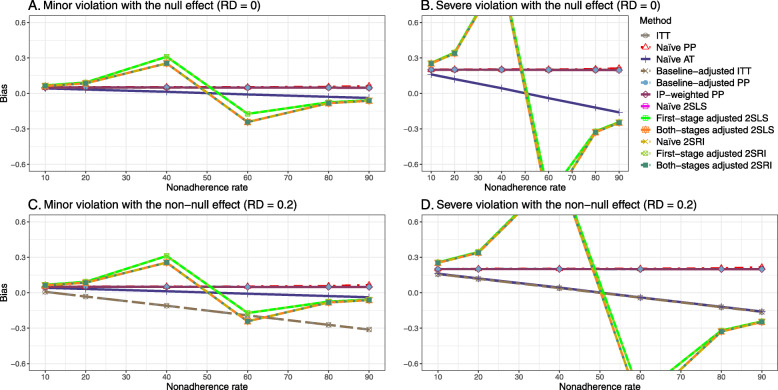
Bias versus the nonadherence rate for the null and non-null effect using DAG 3. Here, violation indicates the violation of the exclusion restriction assumption. The naive and both-stages adjusted 2SLS and 2SRI, and the first-stage adjusted 2SLS and 2SRI methods share the same line as they produce the same amount of bias. The ITT, 2SLS, and 2SRI methods have high bias in some scenarios and thus out of the bound [-0.6,0.6]. Abbreviations: ITT: intention-to-treat, PP: per-protocol, AT: as-treated, IP-weighted PP: inverse probability-weighted per-protocol, 2SLS: two-stage least square, 2SRI: two-stage residual inclusion

**Fig. 21:**
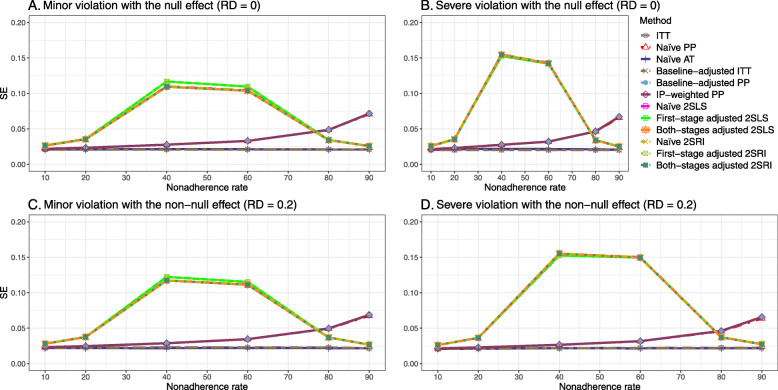
Standard error (SE) versus the nonadherence rate for the null and non-null effect using DAG 3. Here, violation indicates the violation of the exclusion restriction assumption. Abbreviations: ITT: intention-to-treat, PP: per-protocol, AT: as-treated, IP-weighted PP: inverse probability-weighted per-protocol, 2SLS: two-stage least square, 2SRI: two-stage residual inclusion

**Fig. 22:**
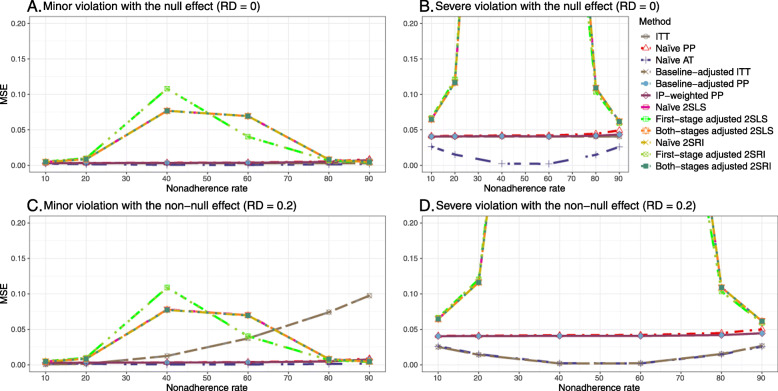
Mean squared error (MSE) versus the nonadherence rate for the null and non-null effect using DAG 3. Here, violation indicates the violation of the exclusion restriction assumption. Abbreviations: ITT: intention-to-treat, PP: per-protocol, AT: as-treated, IP-weighted PP: inverse probability-weighted per-protocol, 2SLS: two-stage least square, 2SRI: two-stage residual inclusion

**Fig. 23:**
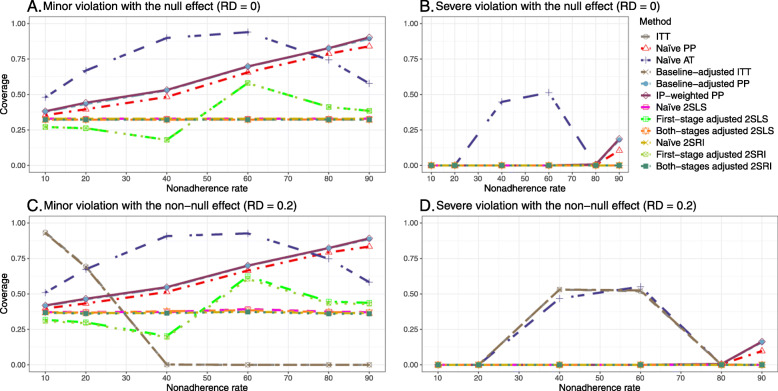
95 percentage coverage probability versus the nonadherence rate for the null and non-null effect using DAG 3. Here, violation indicates the violation of the exclusion restriction assumption. Abbreviations: ITT: intention-to-treat, PP: per-protocol, AT: as-treated, IP-weighted PP: inverse probability-weighted per-protocol, 2SLS: two-stage least square, 2SRI: two-stage residual inclusion

**Fig. 24:**
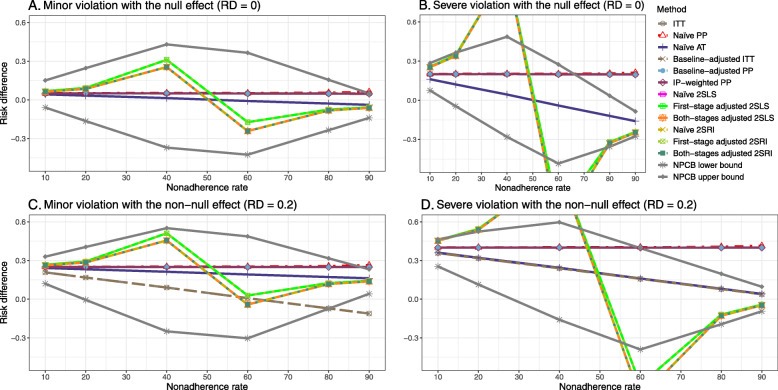
Mean risk difference (RD) versus the nonadherence rate for DAG 3. Here, violation indicates the violation of the exclusion restriction assumption. The grey lines are the lower and upper bound of the NPCB method. The ITT and baseline-adjusted ITT; naive and both-stages adjusted 2SLS and 2SRI superimposed on each other. Abbreviations: RD: risk difference, ITT: intention-to-treat, PP: per-protocol, AT: as-treated, IP-weighted PP: inverse probability-weighted per-protocol, 2SLS: two-stage least square, 2SRI: two-stage residual inclusion, NPCB: nonparametric causal bound

### Appendix DAGs for sensitivity analysis

**Fig. 25:**
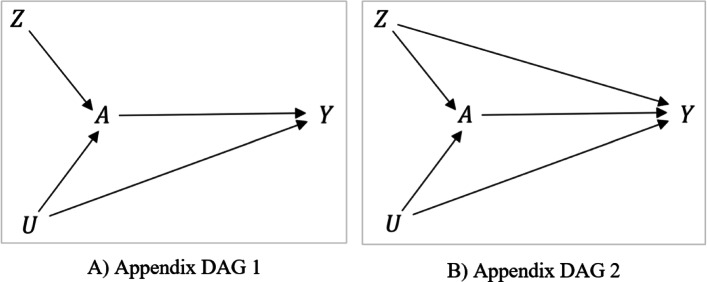
Two simplified versions of causal diagrams representing the simulation mechanisms considered in this study. Appendix DAG 1 is simplified versions of DAG 2 in the main text (unmeasured confounding), and Appendix DAG 2 is the simplified versions of DAG 3 in the main text (exclusion restriction violated). Here, *Z* is the randomization variable, *A* is the treatment, *U* is unmeasured confounders, and *Y* is the outcome

### Results for sensitivity analyses when exclusion restriction satisfied but unmeasured confounding present (Appendix DAG 1)

**Fig. 26:**
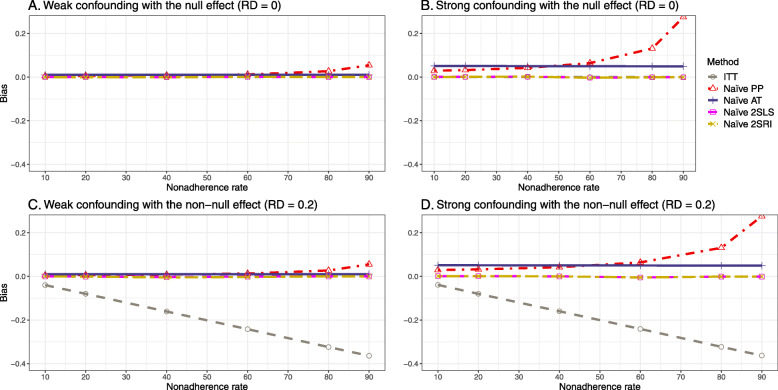
Bias versus the nonadherence rate for the null and non-null effect using Appendix DAG 1. The 2SLS and 2SRI share the same line as they produce the same amount of bias. Abbreviations: RD: risk difference, ITT: intention-to-treat, PP: per-protocol, AT: as-treated, 2SLS: two-stage least square, 2SRI: two-stage residual inclusion

**Fig. 27:**
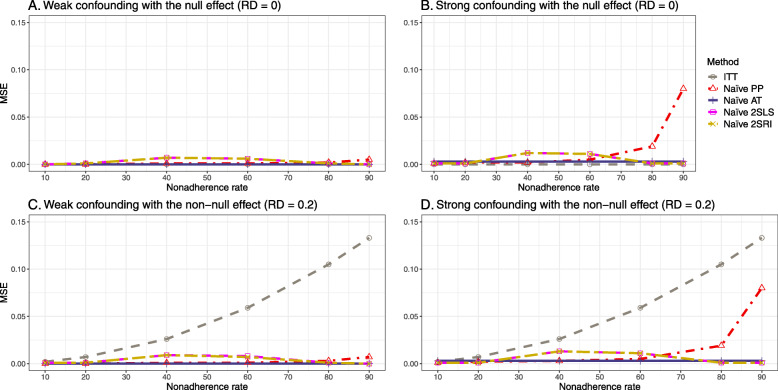
Mean squared error (MSE) versus the nonadherence rate for null and non-null effect using Appendix DAG 1. The 2SLS and 2SRI share the same line as they produce approximately the same amount of MSE. Abbreviations: RD: risk difference, ITT: intention-to-treat, PP: per-protocol, AT: as-treated, 2SLS: two-stage least square, 2SRI: two-stage residual inclusion

**Fig. 28:**
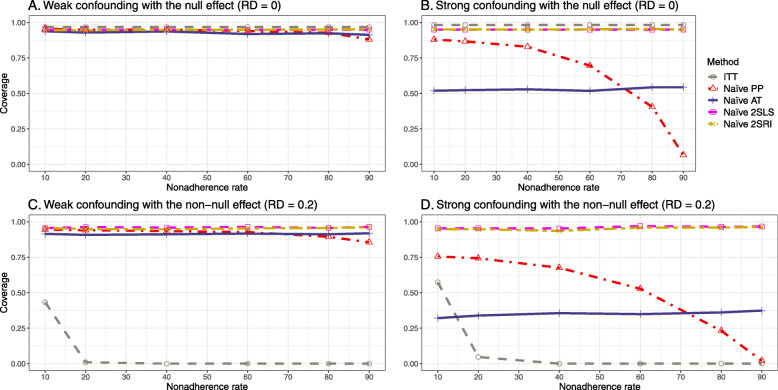
95 percentage coverage probability versus the nonadherence rate for the null and non-null effect using Appendix DAG 1. The 2SLS and 2SRI share the same line as they produce approximately the same coverage probability. Abbreviations: RD: risk difference, ITT: intention-to-treat, PP: per-protocol, AT: as-treated, 2SLS: two-stage least square, 2SRI: two-stage residual inclusion

**Fig. 29:**
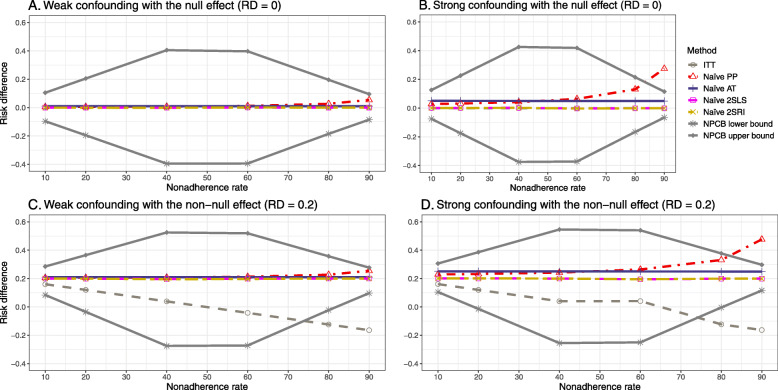
Mean risk difference (RD) versus the nonadherence rate for Appendix DAG 1. The grey lines are the lower and upper bound of the NPCB method. Abbreviations: ITT: intention-to-treat, PP: per-protocol, AT: as-treated, 2SLS: two-stage least square, 2SRI: two-stage residual inclusion, NPCB: nonparametric causal bound

### Results for sensitivity analyses when exclusion restriction violated (Appendix DAG 2)

**Fig. 30:**
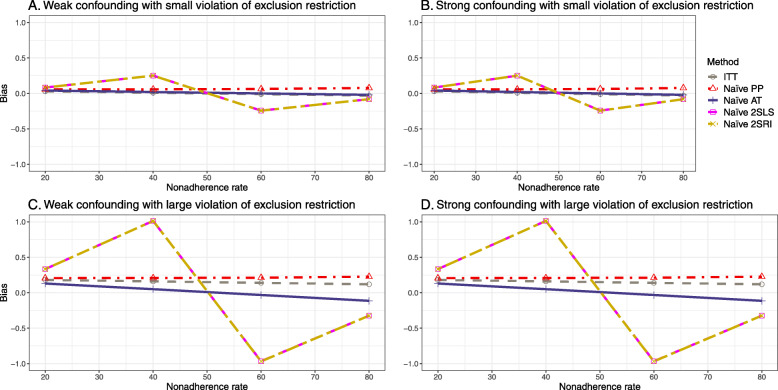
Bias versus the nonadherence rate using Appendix DAG 2. In both scenarios (small/large violation), the target treatment effect is assumed to be 0.05. The 2SLS and 2SRI share the same line as they produce the same amount of bias. Abbreviations: ITT: intention-to-treat, PP: per-protocol, AT: as-treated, 2SLS: two-stage least square, 2SRI: two-stage residual inclusion

### Results for sensitivity analyses with 500 sample size

**Fig. 31:**
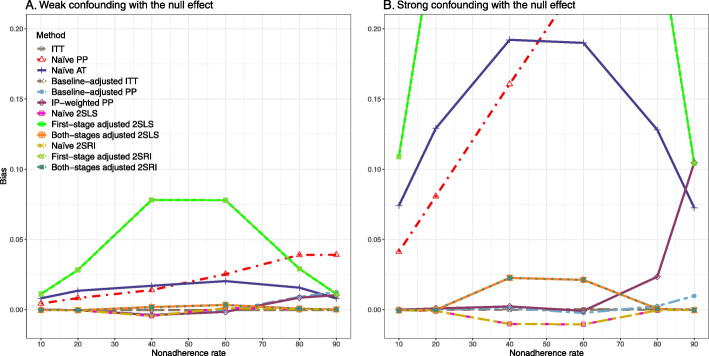
Bias versus the nonadherence rate using DAG 1 for 500 samples. The naive 2SLS and 2SRI share the same line, the first stage adjusted 2SLS and 2SRI share the same line, and both-stages adjusted 2SLS and 2SRI share the same line as they produce the same amount of bias. The first stage adjusted 2SLS and 2SRI, and naive PP produce larger than 0.20 bias for strong unmeasured confounders so that the bias is out of the bound. Abbreviations: ITT: intention-to-treat, PP: per-protocol, AT: as-treated, IP-weighted PP: inverse probability-weighted per-protocol, 2SLS: two-stage least square, 2SRI: two-stage residual inclusion

**Fig. 32:**
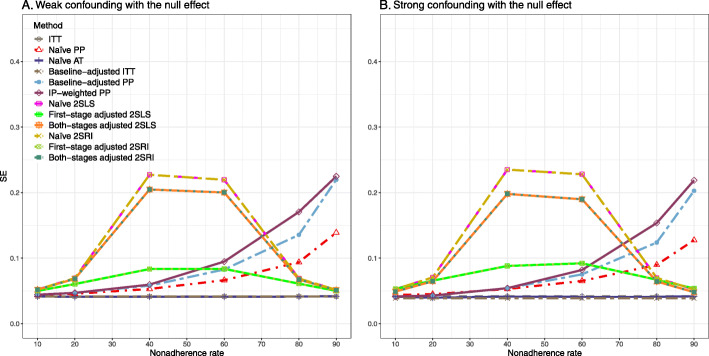
Standard error (SE) versus the nonadherence rate using DAG 1 for 500 samples. The naïve 2SLS and 2SRI share the same line, the first stage adjusted 2SLS and 2SRI share the same line, and both-stages adjusted 2SLS and 2SRI share the same line as they produce approximately the same SE. Abbreviations: ITT: intention-to-treat, PP: per-protocol, AT: as-treated, IP-weighted PP: inverse probability-weighted per-protocol, 2SLS: two-stage least square, 2SRI: two-stage residual inclusion

**Fig. 33:**
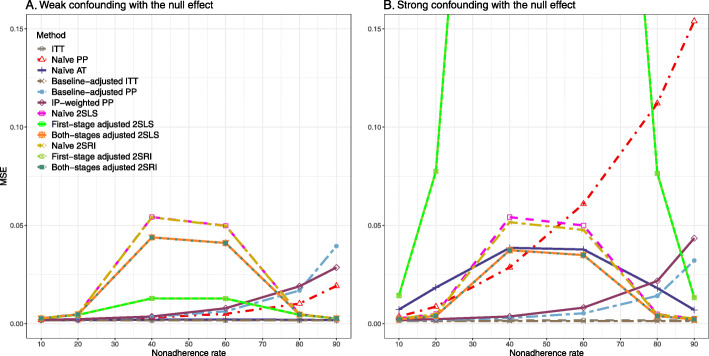
Mean squared error (MSE) versus the nonadherence rate using DAG 1 for 500 samples. The naïve 2SLS and 2SRI share the same line, the first stage adjusted 2SLS and 2SRI share the same line, and both-stages adjusted 2SLS and 2SRI share the same line as they produce approximately the same MSE. The first stage adjusted 2SLS and 2SRI, and naïve PP produce MSEs larger than 0.15 for strong unmeasured confounders so that the MSE is out of the bound [0, 0.15]. Abbreviations: ITT: intention-to-treat, PP: per-protocol, AT: as-treated, IP-weighted PP: inverse probability-weighted per-protocol, 2SLS: two-stage least square, 2SRI: two-stage residual inclusion

**Fig. 34:**
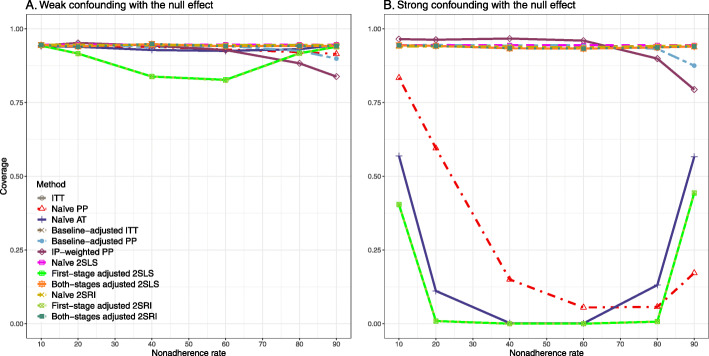
95 percentage coverage probability versus the nonadherence rate using DAG 1 for 500 samples. The naïve 2SLS and 2SRI share the same line, the first-stage adjusted 2SLS and 2SRI share the same line, and both-stages adjusted 2SLS and 2SRI share the same line as they produce approximately the same coverage probability. Abbreviations: ITT: intention-to-treat, PP: per-protocol, AT: as-treated, IP-weighted PP: inverse probability-weighted per-protocol, 2SLS: two-stage least square, 2SRI: two-stage residual inclusion

**Fig. 35:**
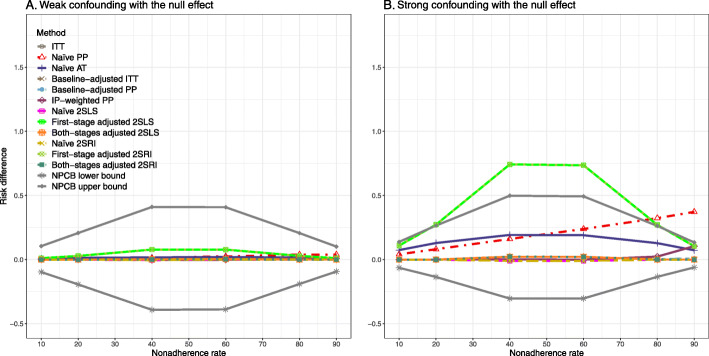
Mean risk difference (RD) versus the nonadherence rate using DAG 1 for 500 samples. The grey lines are the lower and upper bound of the NPCB method. The naïve 2SLS and 2SRI share the same line, the first stage adjusted 2SLS and 2SRI share the same line, and both-stages adjusted 2SLS and 2SRI share the same line as they produce the same RD estimate. Abbreviations: ITT: intention-to-treat, PP: per-protocol, AT: as-treated, IP-weighted PP: inverse probability-weighted per-protocol, 2SLS: two-stage least square, 2SRI: two-stage residual inclusion, NPCB: nonparametric causal bound

**Fig. 36:**
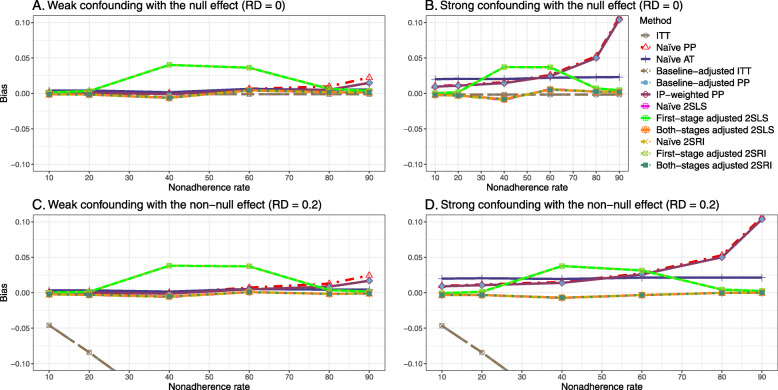
Bias versus the nonadherence rate using DAG 2 for 500 samples. The naïve and both-stages adjusted 2SLS and 2SRI share the same line, and the first stage adjusted 2SLS and 2SRI share the same line as they produce the same amount of bias. Abbreviations: RD: risk difference, ITT: intention-to-treat, PP: per-protocol, AT: as-treated, IP-weighted PP: inverse probability-weighted per-protocol, 2SLS: two-stage least square, 2SRI: two-stage residual inclusion

**Fig. 37:**
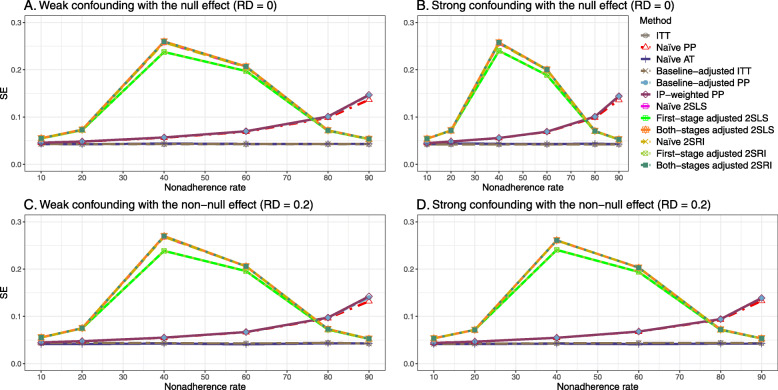
Standard error (SE) versus the nonadherence rate using DAG 2 for 500 samples. The naïve and both-stages adjusted 2SLS and 2SRI share the same line, and the first stage adjusted 2SLS and 2SRI share the same line as they produce approximately the same SE. The PP methods also share approximately the same line. Abbreviations: RD: risk difference, ITT: intention-to-treat, PP: per-protocol, AT: as-treated, IP-weighted PP: inverse probability-weighted per-protocol, 2SLS: two-stage least square, 2SRI: two-stage residual inclusion

**Fig. 38:**
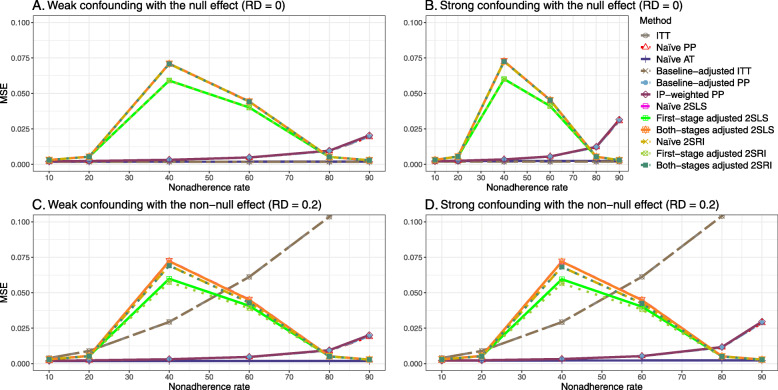
Mean squared error (MSE) versus the nonadherence rate using DAG 2 for 500 samples. The 2SLS and 2SRI share the same line as they produce approximately the same amount of MSE. The ITT and baseline-adjusted ITT produce MSEs larger than 0.10 for the non-null effect scenario so that the MSE is out of the bound [0, 0.10]. Abbreviations: RD: risk difference, ITT: intention-to-treat, PP: per-protocol, AT: as-treated, IP-weighted PP: inverse probability-weighted per-protocol, 2SLS: two-stage least square, 2SRI: two-stage residual inclusion

**Fig. 39:**
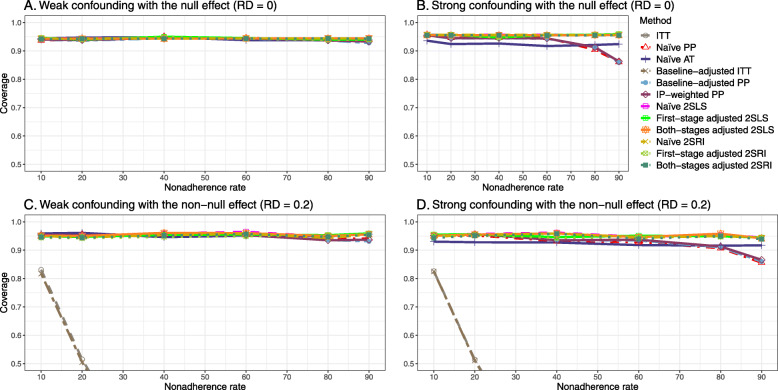
95 percentage coverage probability versus the nonadherence rate for using DAG 2 for 500 samples. The naive and both-stages adjusted 2SLS and 2SRI share the same line as they produce approximately the same coverage probability. The coverage probability is out of bound for the ITT methods with the non-null effect. Abbreviations: RD: risk difference, ITT: intention-to-treat, PP: per-protocol, AT: as-treated, IP-weighted PP: inverse probability-weighted per-protocol, 2SLS: two-stage least square, 2SRI: two-stage residual inclusion

**Fig. 40:**
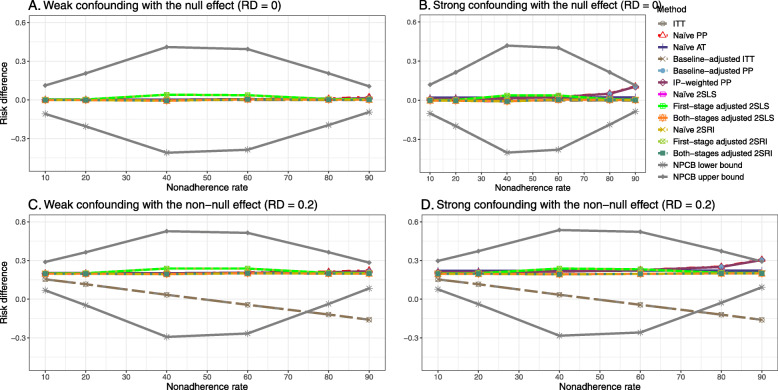
Mean risk difference (RD) versus the nonadherence rate for using DAG 2 for 500 samples. The grey lines are the lower and upper bound of the NPCB method. The ITT and baseline-adjusted ITT superimposed on each other. Abbreviations: RD: risk difference, ITT: intention-to-treat, PP: per-protocol, AT: as-treated, IP-weighted PP: inverse probability-weighted per-protocol, 2SLS: two-stage least square, 2SRI: two-stage residual inclusion, NPCB: nonparametric causal bound

**Fig. 41:**
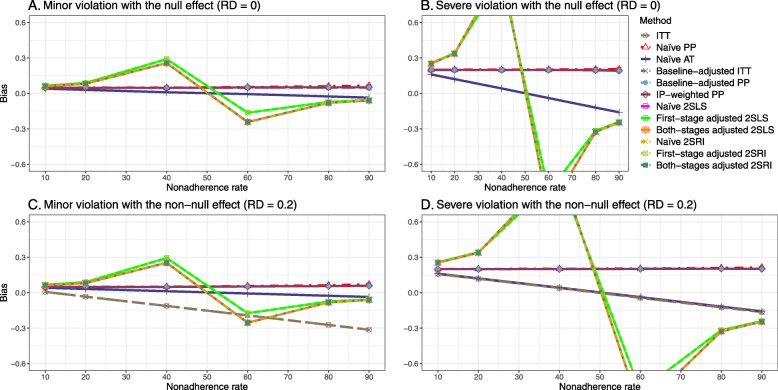
Bias versus the nonadherence rate using DAG 3 for 500 samples. Here, violation indicates the violation of the exclusion restriction assumption. The both-stages adjusted 2SLS and 2SRI methods share approximately the same line. These methods have high biases when the exclusion restriction violation is severe and thus out of the bound. The biases for ITT methods are also out of the bound for the non-null effect. Abbreviations: ITT: intention-to-treat, PP: per-protocol, AT: as-treated, IP-weighted PP: inverse probability-weighted per-protocol, 2SLS: two-stage least square, 2SRI: two-stage residual inclusion

**Fig. 42:**
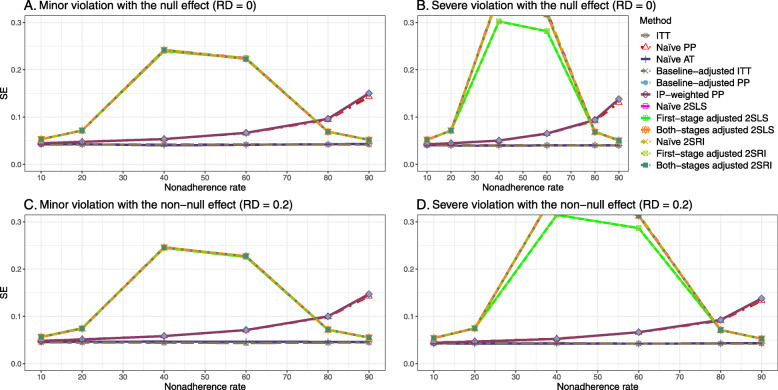
Standard error (SE) versus the nonadherence rate using DAG 3 for 500 samples. Here, violation indicates the violation of the exclusion restriction assumption. Abbreviations: ITT: intention-to-treat, PP: per-protocol, AT: as-treated, IP-weighted PP: inverse probability-weighted per-protocol, 2SLS: two-stage least square, 2SRI: two-stage residual inclusion

**Fig. 43:**
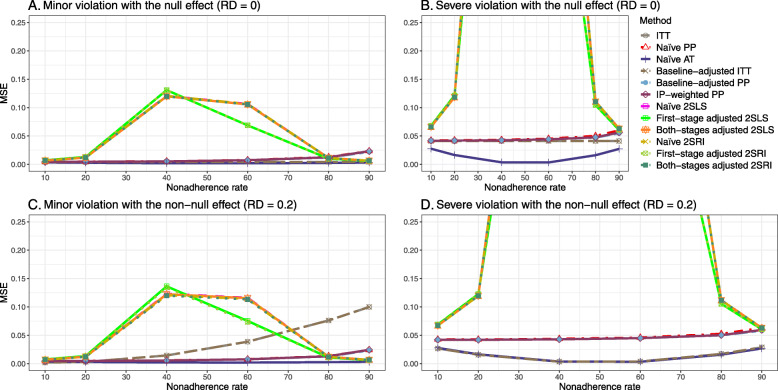
Mean squared error (MSE) versus the nonadherence rate using DAG 3 for 500 samples. Here, violation indicates the violation of the exclusion restriction assumption. Abbreviations: ITT: intention-to-treat, PP: per-protocol, AT: as-treated, IP-weighted PP: inverse probability-weighted per-protocol, 2SLS: two-stage least square, 2SRI: two-stage residual inclusion

**Fig. 44:**
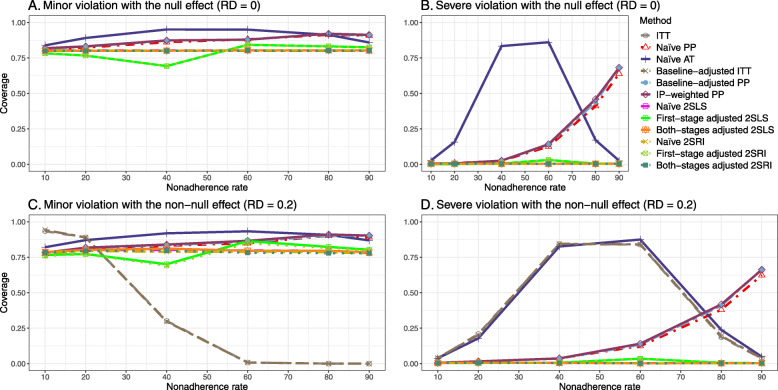
95 percentage coverage probability versus the nonadherence rate using DAG 3 for 500 samples. Here, violation indicates the violation of the exclusion restriction assumption. Abbreviations: ITT: intention-to-treat, PP: per-protocol, AT: as-treated, IP-weighted PP: inverse probability-weighted per-protocol, 2SLS: two-stage least square, 2SRI: two-stage residual inclusion

**Fig. 45:**
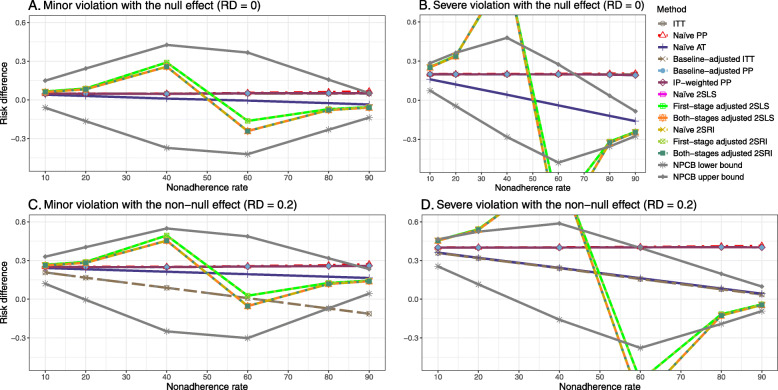
Mean risk difference (RD) versus the nonadherence rate using DAG 3 for 500 samples. Here, violation indicates the violation of the exclusion restriction assumption. The grey lines are the lower and upper bound of the NPCB method. The ITT and baseline-adjusted ITT; naive and both-stages adjusted 2SLS and 2SRI superimposed on each other. Abbreviations: RD: risk difference, ITT: intention-to-treat, PP: per-protocol, AT: as-treated, IP-weighted PP: inverse probability-weighted per-protocol, 2SLS: two-stage least square, 2SRI: two-stage residual inclusion, NPCB: nonparametric causal bound

### Present study’s recommendation

**Fig. 46:**
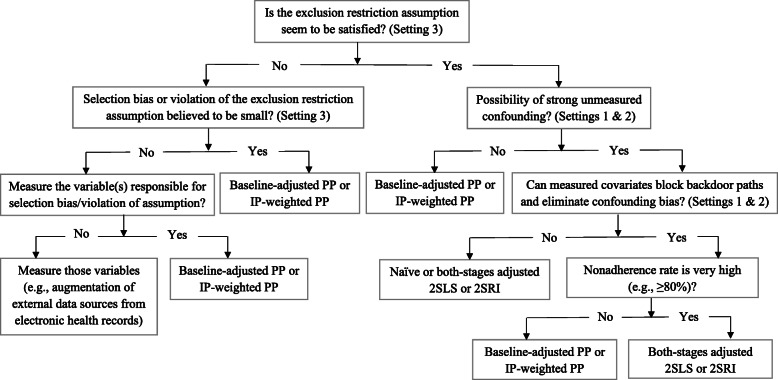
Flow chart showing the conclusion or recommendation from the present study. Abbreviations: PP: per-protocol, IP-weighted PP: inverse probability-weighted per-protocol, 2SLS: two-stage least square, 2SRI: two-stage residual inclusion

## Appendix D

This section is for the parameterization of each three data-generating processes in this study. Appendix D Tables 5–7 show the parameters for the simulation settings 1-3 outlined in DAGs 1-3, respectively. In each table, we have the same set of parameters: *α*_0_ determines the nonadherence rate, *α*_1_ is the coefficient for *Z*-*A* association, *α*_2_ is the coefficient for *L*_1_-*A* association, *α*_3_ is the coefficient for *L*_2_-*A* association, and *α*_4_ is the coefficient for *U*-*A* association. Also, *θ*_0_ determines the event rate, *θ*_1_ is treatment effect of interest (i.e., the coefficient for *A*-*Y* association), *θ*_2_ is the coefficient for *L*_1_-*Y* association, *θ*_3_ is the coefficient for *L*_2_-*Y* association, *θ*_4_ is the coefficient for *U*-*Y* association, and *θ*_5_ determines the *Z*-*Y* association.

**Table 5:** Parameterization of simulation for data generating process for simulation setting 1 outlines in DAG 1 (exclusion restriction satisfied and no unmeasured confounding)

#	Arm	*α*_0_	*α*_1_	*α*_2_	*α*_3_	*α*_4_	Nonadherence	*θ*_0_	*θ*_1_	*θ*_2_	*θ*_3_	*θ*_4_	*θ*_5_
1	Z = 1	0.72	0.6	0.4	0.35	0	10	-1	0	0	0	0.5	0
	Z = 0	-4.06	0.6	0.4	0.35	0	10	-1	0	0	0	0.5	0
2	Z = 1	-0.23	0.6	0.4	0.35	0	20	-1	0	0	0	0.5	0
	Z = 0	-3.14	0.6	0.4	0.35	0	20	-1	0	0	0	0.5	0
3	Z = 1	-1.47	0.6	0.4	0.35	0	40	-1	0	0	0	0.5	0
	Z = 0	-1.92	0.6	0.4	0.35	0	40	-1	0	0	0	0.5	0
4	Z = 1	-2.52	0.6	0.4	0.35	0	60	-1	0	0	0	0.5	0
	Z = 0	-0.85	0.6	0.4	0.35	0	60	-1	0	0	0	0.5	0
5	Z = 1	-3.76	0.6	0.4	0.35	0	80	-1	0	0	0	0.5	0
	Z = 0	0.39	0.6	0.4	0.35	0	80	-1	0	0	0	0.5	0
6	Z = 1	-4.72	0.6	0.4	0.35	0	90	-1	0	0	0	0.5	0
	Z = 0	1.35	0.6	0.4	0.35	0	90	-1	0	0	0	0.5	0
7	Z = 1	0.72	0.6	0.4	0.35	0	10	-5.5	0	0	0	8	0
	Z = 0	-4.06	0.6	0.4	0.35	0	10	-5.5	0	0	0	8	0
8	Z = 1	-0.23	0.6	0.4	0.35	0	20	-5.5	0	0	0	8	0
	Z = 0	-3.14	0.6	0.4	0.35	0	20	-5.5	0	0	0	8	0
9	Z = 1	-1.47	0.6	0.4	0.35	0	40	-5.5	0	0	0	8	0
	Z = 0	-1.92	0.6	0.4	0.35	0	40	-5.5	0	0	0	8	0
10	Z = 1	-2.52	0.6	0.4	0.35	0	60	-5.5	0	0	0	8	0
	Z = 0	-0.85	0.6	0.4	0.35	0	60	-5.5	0	0	0	8	0
11	Z = 1	-3.76	0.6	0.4	0.35	0	80	-5.5	0	0	0	8	0
	Z = 0	0.39	0.6	0.4	0.35	0	80	-5.5	0	0	0	8	0
12	Z = 1	-4.72	0.6	0.4	0.35	0	90	-5.5	0	0	0	8	0
	Z = 0	1.35	0.6	0.4	0.35	0	90	-5.5	0	0	0	8	0

**Table 6:** Parameterization of simulation for data generating process for simulation setting 2 outlines in DAG 2 (Exclusion restriction satisfied, unmeasured confounding present)

#	Arm	*α*_0_	*α*_1_	*α*_2_	*α*_3_	*α*_4_	Nonadherence	*θ*_0_	*θ*_1_	*θ*_2_	*θ*_3_	*θ*_4_	*θ*_5_
1	Z = 1	0.55	0.25	0.02	0.04	0.05	11	0.35	-0.2	0.02	0.05	0.05	0
	Z = 0	0.02	0.25	0.02	0.04	0.05	11	0.35	-0.2	0.02	0.05	0.05	0
2	Z = 1	0.46	0.25	0.02	0.04	0.05	20	0.35	-0.2	0.02	0.05	0.05	0
	Z = 0	0.12	0.25	0.02	0.04	0.05	21	0.35	-0.2	0.02	0.05	0.05	0
3	Z = 1	0.25	0.25	0.02	0.04	0.05	41	0.35	-0.2	0.02	0.05	0.05	0
	Z = 0	0.32	0.25	0.02	0.04	0.05	41	0.35	-0.2	0.02	0.05	0.05	0

**Table 6:** Parameterization of simulation for data generating process for simulation setting 2 outlines in DAG 2 (Exclusion restriction satisfied, unmeasured confounding present) (*Continued*)

#	Arm	*α*_0_	*α*_1_	*α*_2_	*α*_3_	*α*_4_	Nonadherence	*θ*_0_	*θ*_1_	*θ*_2_	*θ*_3_	*θ*_4_	*θ*_5_
4	Z = 1	0.05	0.25	0.02	0.04	0.05	61	0.35	-0.2	0.02	0.05	0.05	0
	Z = 0	0.52	0.25	0.02	0.04	0.05	61	0.35	-0.2	0.02	0.05	0.05	0
5	Z = 1	-0.15	0.25	0.02	0.04	0.05	81	0.35	-0.2	0.02	0.05	0.05	0
	Z = 0	0.7	0.25	0.02	0.04	0.05	80	0.35	-0.2	0.02	0.05	0.05	0
6	Z = 1	-0.25	0.25	0.02	0.04	0.05	91	0.35	-0.2	0.02	0.05	0.05	0
	Z = 0	0.8	0.25	0.02	0.04	0.05	89	0.35	-0.2	0.02	0.05	0.05	0
7	Z = 1	0.55	0.25	0.02	0.04	0.05	11	0.2	-0.2	0.02	0.05	0.4	0
	Z = 0	0.02	0.25	0.02	0.04	0.05	11	0.2	-0.2	0.02	0.05	0.4	0
8	Z = 1	0.46	0.25	0.02	0.04	0.05	20	0.2	-0.2	0.02	0.05	0.4	0
	Z = 0	0.12	0.25	0.02	0.04	0.05	21	0.2	-0.2	0.02	0.05	0.4	0
9	Z = 1	0.25	0.25	0.02	0.04	0.05	41	0.2	-0.2	0.02	0.05	0.4	0
	Z = 0	0.32	0.25	0.02	0.04	0.05	41	0.2	-0.2	0.02	0.05	0.4	0
10	Z = 1	0.05	0.25	0.02	0.04	0.05	61	0.2	-0.2	0.02	0.05	0.4	0
	Z = 0	0.52	0.25	0.02	0.04	0.05	61	0.2	-0.2	0.02	0.05	0.4	0
11	Z = 1	-0.15	0.25	0.02	0.04	0.05	81	0.2	-0.2	0.02	0.05	0.4	0
	Z = 0	0.7	0.25	0.02	0.04	0.05	80	0.2	-0.2	0.02	0.05	0.4	0
12	Z = 1	-0.25	0.25	0.02	0.04	0.05	91	0.2	-0.2	0.02	0.05	0.4	0
	Z = 0	0.8	0.25	0.02	0.04	0.05	89	0.2	-0.2	0.02	0.05	0.4	0
13	Z = 1	0.55	0.25	0.02	0.04	0.05	11	0.3	-0.05	0.02	0.05	0.05	0
	Z = 0	0.02	0.25	0.02	0.04	0.05	11	0.3	-0.05	0.02	0.05	0.05	0
14	Z = 1	0.46	0.25	0.02	0.04	0.05	20	0.3	-0.05	0.02	0.05	0.05	0
	Z = 0	0.12	0.25	0.02	0.04	0.05	21	0.3	-0.05	0.02	0.05	0.05	0
15	Z = 1	0.25	0.25	0.02	0.04	0.05	41	0.3	-0.05	0.02	0.05	0.05	0
	Z = 0	0.32	0.25	0.02	0.04	0.05	41	0.3	-0.05	0.02	0.05	0.05	0
16	Z = 1	0.05	0.25	0.02	0.04	0.05	61	0.3	-0.05	0.02	0.05	0.05	0
	Z = 0	0.52	0.25	0.02	0.04	0.05	61	0.3	-0.05	0.02	0.05	0.05	0
17	Z = 1	-0.15	0.25	0.02	0.04	0.05	81	0.3	-0.05	0.02	0.05	0.05	0
	Z = 0	0.7	0.25	0.02	0.04	0.05	80	0.3	-0.05	0.02	0.05	0.05	0
18	Z = 1	-0.25	0.25	0.02	0.04	0.05	91	0.3	-0.05	0.02	0.05	0.05	0
	Z = 0	0.8	0.25	0.02	0.04	0.05	89	0.3	-0.05	0.02	0.05	0.05	0
19	Z = 1	0.55	0.25	0.02	0.04	0.05	11	0.15	-0.05	0.02	0.05	0.4	0
	Z = 0	0.02	0.25	0.02	0.04	0.05	11	0.15	-0.05	0.02	0.05	0.4	0
20	Z = 1	0.46	0.25	0.02	0.04	0.05	20	0.15	-0.05	0.02	0.05	0.4	0
	Z = 0	0.12	0.25	0.02	0.04	0.05	21	0.15	-0.05	0.02	0.05	0.4	0
21	Z = 1	0.25	0.25	0.02	0.04	0.05	41	0.15	-0.05	0.02	0.05	0.4	0
	Z = 0	0.32	0.25	0.02	0.04	0.05	41	0.15	-0.05	0.02	0.05	0.4	0
22	Z = 1	0.05	0.25	0.02	0.04	0.05	61	0.15	-0.05	0.02	0.05	0.4	0
	Z = 0	0.52	0.25	0.02	0.04	0.05	61	0.15	-0.05	0.02	0.05	0.4	0
23	Z = 1	-0.15	0.25	0.02	0.04	0.05	81	0.15	-0.05	0.02	0.05	0.4	0
	Z = 0	0.7	0.25	0.02	0.04	0.05	80	0.15	-0.05	0.02	0.05	0.4	0
24	Z = 1	-0.25	0.25	0.02	0.04	0.05	91	0.15	-0.05	0.02	0.05	0.4	0
	Z = 0	0.8	0.25	0.02	0.04	0.05	89	0.15	-0.05	0.02	0.05	0.4	0

**Table 6:** Parameterization of simulation for data generating process for simulation setting 2 outlines in DAG 2 (Exclusion restriction satisfied, unmeasured confounding present) (*Continued*)

#	Arm	*α*_0_	*α*_1_	*α*_2_	*α*_3_	*α*_4_	Nonadherence	*θ*_0_	*θ*_1_	*θ*_2_	*θ*_3_	*θ*_4_	*θ*_5_
25	Z = 1	0.55	0.25	0.02	0.04	0.05	11	0.28	0	0.02	0.05	0.05	0
	Z = 0	0.02	0.25	0.02	0.04	0.05	11	0.28	0	0.02	0.05	0.05	0
26	Z = 1	0.46	0.25	0.02	0.04	0.05	20	0.28	0	0.02	0.05	0.05	0
	Z = 0	0.12	0.25	0.02	0.04	0.05	21	0.28	0	0.02	0.05	0.05	0
27	Z = 1	0.25	0.25	0.02	0.04	0.05	41	0.28	0	0.02	0.05	0.05	0
	Z = 0	0.32	0.25	0.02	0.04	0.05	41	0.28	0	0.02	0.05	0.05	0
28	Z = 1	0.05	0.25	0.02	0.04	0.05	61	0.28	0	0.02	0.05	0.05	0
	Z = 0	0.52	0.25	0.02	0.04	0.05	61	0.28	0	0.02	0.05	0.05	0
29	Z = 1	-0.15	0.25	0.02	0.04	0.05	81	0.28	0	0.02	0.05	0.05	0
	Z = 0	0.7	0.25	0.02	0.04	0.05	80	0.28	0	0.02	0.05	0.05	0
30	Z = 1	-0.25	0.25	0.02	0.04	0.05	91	0.28	0	0.02	0.05	0.05	0
	Z = 0	0.8	0.25	0.02	0.04	0.05	89	0.28	0	0.02	0.05	0.05	0
31	Z = 1	0.55	0.25	0.02	0.04	0.05	11	0.13	0	0.02	0.05	0.4	0
	Z = 0	0.02	0.25	0.02	0.04	0.05	11	0.13	0	0.02	0.05	0.4	0
32	Z = 1	0.46	0.25	0.02	0.04	0.05	20	0.13	0	0.02	0.05	0.4	0
	Z = 0	0.12	0.25	0.02	0.04	0.05	21	0.13	0	0.02	0.05	0.4	0
33	Z = 1	0.25	0.25	0.02	0.04	0.05	41	0.13	0	0.02	0.05	0.4	0
	Z = 0	0.32	0.25	0.02	0.04	0.05	41	0.13	0	0.02	0.05	0.4	0
34	Z = 1	0.05	0.25	0.02	0.04	0.05	61	0.13	0	0.02	0.05	0.4	0
	Z = 0	0.52	0.25	0.02	0.04	0.05	61	0.13	0	0.02	0.05	0.4	0
35	Z = 1	-0.15	0.25	0.02	0.04	0.05	81	0.13	0	0.02	0.05	0.4	0
	Z = 0	0.7	0.25	0.02	0.04	0.05	80	0.13	0	0.02	0.05	0.4	0
36	Z = 1	-0.25	0.25	0.02	0.04	0.05	91	0.13	0	0.02	0.05	0.4	0
	Z = 0	0.8	0.25	0.02	0.04	0.05	89	0.13	0	0.02	0.05	0.4	0
37	Z = 1	0.55	0.25	0.02	0.04	0.05	11	0.25	0.05	0.02	0.05	0.05	0
	Z = 0	0.02	0.25	0.02	0.04	0.05	11	0.25	0.05	0.02	0.05	0.05	0
38	Z = 1	0.46	0.25	0.02	0.04	0.05	20	0.25	0.05	0.02	0.05	0.05	0
	Z = 0	0.12	0.25	0.02	0.04	0.05	21	0.25	0.05	0.02	0.05	0.05	0
39	Z = 1	0.25	0.25	0.02	0.04	0.05	41	0.25	0.05	0.02	0.05	0.05	0
	Z = 0	0.32	0.25	0.02	0.04	0.05	41	0.25	0.05	0.02	0.05	0.05	0
40	Z = 1	0.05	0.25	0.02	0.04	0.05	61	0.25	0.05	0.02	0.05	0.05	0
	Z = 0	0.52	0.25	0.02	0.04	0.05	61	0.25	0.05	0.02	0.05	0.05	0
41	Z = 1	-0.15	0.25	0.02	0.04	0.05	81	0.25	0.05	0.02	0.05	0.05	0
	Z = 0	0.7	0.25	0.02	0.04	0.05	80	0.25	0.05	0.02	0.05	0.05	0
42	Z = 1	-0.25	0.25	0.02	0.04	0.05	91	0.25	0.05	0.02	0.05	0.05	0
	Z = 0	0.8	0.25	0.02	0.04	0.05	89	0.25	0.05	0.02	0.05	0.05	0
43	Z = 1	0.55	0.25	0.02	0.04	0.05	11	0.1	0.05	0.02	0.05	0.4	0
	Z = 0	0.02	0.25	0.02	0.04	0.05	11	0.1	0.05	0.02	0.05	0.4	0
44	Z = 1	0.46	0.25	0.02	0.04	0.05	20	0.1	0.05	0.02	0.05	0.4	0
	Z = 0	0.12	0.25	0.02	0.04	0.05	21	0.1	0.05	0.02	0.05	0.4	0
45	Z = 1	0.25	0.25	0.02	0.04	0.05	41	0.1	0.05	0.02	0.05	0.4	0
	Z = 0	0.32	0.25	0.02	0.04	0.05	41	0.1	0.05	0.02	0.05	0.4	0

**Table 6:** Parameterization of simulation for data generating process for simulation setting 2 outlines in DAG 2 (Exclusion restriction satisfied, unmeasured confounding present) (*Continued*)

#	Arm	*α*_0_	*α*_1_	*α*_2_	*α*_3_	*α*_4_	Nonadherence	*θ*_0_	*θ*_1_	*θ*_2_	*θ*_3_	*θ*_4_	*θ*_5_
46	Z = 1	0.05	0.25	0.02	0.04	0.05	61	0.1	0.05	0.02	0.05	0.4	0
	Z = 0	0.52	0.25	0.02	0.04	0.05	61	0.1	0.05	0.02	0.05	0.4	0
47	Z = 1	-0.15	0.25	0.02	0.04	0.05	81	0.1	0.05	0.02	0.05	0.4	0
	Z = 0	0.7	0.25	0.02	0.04	0.05	80	0.1	0.05	0.02	0.05	0.4	0
48	Z = 1	-0.25	0.25	0.02	0.04	0.05	91	0.1	0.05	0.02	0.05	0.4	0
	Z = 0	0.8	0.25	0.02	0.04	0.05	89	0.1	0.05	0.02	0.05	0.4	0
49	Z = 1	0.55	0.25	0.02	0.04	0.05	11	0.2	0.2	0.02	0.05	0.05	0
	Z = 0	0.02	0.25	0.02	0.04	0.05	11	0.2	0.2	0.02	0.05	0.05	0
50	Z = 1	0.46	0.25	0.02	0.04	0.05	20	0.2	0.2	0.02	0.05	0.05	0
	Z = 0	0.12	0.25	0.02	0.04	0.05	21	0.2	0.2	0.02	0.05	0.05	0
51	Z = 1	0.25	0.25	0.02	0.04	0.05	41	0.2	0.2	0.02	0.05	0.05	0
	Z = 0	0.32	0.25	0.02	0.04	0.05	41	0.2	0.2	0.02	0.05	0.05	0
52	Z = 1	0.05	0.25	0.02	0.04	0.05	61	0.2	0.2	0.02	0.05	0.05	0
	Z = 0	0.52	0.25	0.02	0.04	0.05	61	0.2	0.2	0.02	0.05	0.05	0
53	Z = 1	-0.15	0.25	0.02	0.04	0.05	81	0.2	0.2	0.02	0.05	0.05	0
	Z = 0	0.7	0.25	0.02	0.04	0.05	80	0.2	0.2	0.02	0.05	0.05	0
54	Z = 1	-0.25	0.25	0.02	0.04	0.05	91	0.2	0.2	0.02	0.05	0.05	0
	Z = 0	0.8	0.25	0.02	0.04	0.05	89	0.2	0.2	0.02	0.05	0.05	0
55	Z = 1	0.55	0.25	0.02	0.04	0.05	11	0.02	0.2	0.02	0.05	0.4	0
	Z = 0	0.02	0.25	0.02	0.04	0.05	11	0.02	0.2	0.02	0.05	0.4	0
56	Z = 1	0.46	0.25	0.02	0.04	0.05	20	0.02	0.2	0.02	0.05	0.4	0
	Z = 0	0.12	0.25	0.02	0.04	0.05	21	0.02	0.2	0.02	0.05	0.4	0
57	Z = 1	0.25	0.25	0.02	0.04	0.05	41	0.02	0.2	0.02	0.05	0.4	0
	Z = 0	0.32	0.25	0.02	0.04	0.05	41	0.02	0.2	0.02	0.05	0.4	0
58	Z = 1	0.05	0.25	0.02	0.04	0.05	61	0.02	0.2	0.02	0.05	0.4	0
	Z = 0	0.52	0.25	0.02	0.04	0.05	61	0.02	0.2	0.02	0.05	0.4	0
59	Z = 1	-0.15	0.25	0.02	0.04	0.05	81	0.02	0.2	0.02	0.05	0.4	0
	Z = 0	0.7	0.25	0.02	0.04	0.05	80	0.02	0.2	0.02	0.05	0.4	0
60	Z = 1	-0.25	0.25	0.02	0.04	0.05	91	0.02	0.2	0.02	0.05	0.4	0
	Z = 0	0.8	0.25	0.02	0.04	0.05	89	0.02	0.2	0.02	0.05	0.4	0

**Table 7:** Parameterization of simulation for data generating process for simulation setting 3 outlines in DAG 3 (Exclusion restriction violated)

#	Arm	*α*_0_	*α*_1_	*α*_2_	*α*_3_	*α*_4_	Nonadherence	*θ*_0_	*θ*_1_	*θ*_2_	*θ*_3_	*θ*_4_	*θ*_5_
1	Z = 1	0.86	0	0.01	0.04	0	10	0.2	0	0.03	0.1	0.05	0.05
	Z = 0	0.06	0	0.01	0.04	0	10	0.2	0	0.03	0.1	0.05	0.05
2	Z = 1	0.76	0	0.01	0.04	0	20	0.2	0	0.03	0.1	0.05	0.05
	Z = 0	0.16	0	0.01	0.04	0	20	0.2	0	0.03	0.1	0.05	0.05
3	Z = 1	0.56	0	0.01	0.04	0	41	0.2	0	0.03	0.1	0.05	0.05
	Z = 0	0.36	0	0.01	0.04	0	40	0.2	0	0.03	0.1	0.05	0.05
4	Z = 1	0.36	0	0.01	0.04	0	60	0.2	0	0.03	0.1	0.05	0.05
	Z = 0	0.57	0	0.01	0.04	0	60	0.2	0	0.03	0.1	0.05	0.05

**Table 7:** Parameterization of simulation for data generating process for simulation setting 3 outlines in DAG 3 (Exclusion restriction violated) (*Continued*)

#	Arm	*α*_0_	*α*_1_	*α*_2_	*α*_3_	*α*_4_	Nonadherence	*θ*_0_	*θ*_1_	*θ*_2_	*θ*_3_	*θ*_4_	*θ*_5_
5	Z = 1	0.16	0	0.01	0.04	0	80	0.2	0	0.03	0.1	0.05	0.05
	Z = 0	0.77	0	0.01	0.04	0	80	0.2	0	0.03	0.1	0.05	0.05
6	Z = 1	0.06	0	0.01	0.04	0	90	0.2	0	0.03	0.1	0.05	0.05
	Z = 0	0.86	0	0.01	0.04	0	90	0.2	0	0.03	0.1	0.05	0.05
7	Z = 1	0.86	0	0.01	0.04	0	10	0.1	0	0.03	0.1	0.05	0.2
	Z = 0	0.06	0	0.01	0.04	0	10	0.1	0	0.03	0.1	0.05	0.2
8	Z = 1	0.76	0	0.01	0.04	0	20	0.1	0	0.03	0.1	0.05	0.2
	Z = 0	0.16	0	0.01	0.04	0	20	0.1	0	0.03	0.1	0.05	0.2
9	Z = 1	0.56	0	0.01	0.04	0	41	0.1	0	0.03	0.1	0.05	0.2
	Z = 0	0.36	0	0.01	0.04	0	40	0.1	0	0.03	0.1	0.05	0.2
10	Z = 1	0.36	0	0.01	0.04	0	60	0.1	0	0.03	0.1	0.05	0.2
	Z = 0	0.57	0	0.01	0.04	0	60	0.1	0	0.03	0.1	0.05	0.2
11	Z = 1	0.16	0	0.01	0.04	0	80	0.1	0	0.03	0.1	0.05	0.2
	Z = 0	0.77	0	0.01	0.04	0	80	0.1	0	0.03	0.1	0.05	0.2
12	Z = 1	0.06	0	0.01	0.04	0	90	0.1	0	0.03	0.1	0.05	0.2
	Z = 0	0.86	0	0.01	0.04	0	90	0.1	0	0.03	0.1	0.05	0.2
13	Z = 1	0.86	0	0.01	0.04	0	10	0.2	0.2	0.03	0.1	0.05	0.05
	Z = 0	0.06	0	0.01	0.04	0	10	0.2	0.2	0.03	0.1	0.05	0.05
14	Z = 1	0.76	0	0.01	0.04	0	20	0.2	0.2	0.03	0.1	0.05	0.05
	Z = 0	0.16	0	0.01	0.04	0	20	0.2	0.2	0.03	0.1	0.05	0.05
15	Z = 1	0.56	0	0.01	0.04	0	41	0.2	0.2	0.03	0.1	0.05	0.05
	Z = 0	0.36	0	0.01	0.04	0	40	0.2	0.2	0.03	0.1	0.05	0.05
16	Z = 1	0.36	0	0.01	0.04	0	60	0.2	0.2	0.03	0.1	0.05	0.05
	Z = 0	0.57	0	0.01	0.04	0	60	0.2	0.2	0.03	0.1	0.05	0.05
17	Z = 1	0.16	0	0.01	0.04	0	80	0.2	0.2	0.03	0.1	0.05	0.05
	Z = 0	0.77	0	0.01	0.04	0	80	0.2	0.2	0.03	0.1	0.05	0.05
18	Z = 1	0.06	0	0.01	0.04	0	90	0.2	0.2	0.03	0.1	0.05	0.05
	Z = 0	0.86	0	0.01	0.04	0	90	0.2	0.2	0.03	0.1	0.05	0.05
19	Z = 1	0.86	0	0.01	0.04	0	10	0.1	0.2	0.03	0.1	0.05	0.2
	Z = 0	0.06	0	0.01	0.04	0	10	0.1	0.2	0.03	0.1	0.05	0.2
20	Z = 1	0.76	0	0.01	0.04	0	20	0.1	0.2	0.03	0.1	0.05	0.2
	Z = 0	0.16	0	0.01	0.04	0	20	0.1	0.2	0.03	0.1	0.05	0.2
21	Z = 1	0.56	0	0.01	0.04	0	41	0.1	0.2	0.03	0.1	0.05	0.2
	Z = 0	0.36	0	0.01	0.04	0	40	0.1	0.2	0.03	0.1	0.05	0.2
22	Z = 1	0.36	0	0.01	0.04	0	60	0.1	0.2	0.03	0.1	0.05	0.2
	Z = 0	0.57	0	0.01	0.04	0	60	0.1	0.2	0.03	0.1	0.05	0.2
23	Z = 1	0.16	0	0.01	0.04	0	80	0.1	0.2	0.03	0.1	0.05	0.2
	Z = 0	0.77	0	0.01	0.04	0	80	0.1	0.2	0.03	0.1	0.05	0.2
24	Z = 1	0.06	0	0.01	0.04	0	90	0.1	0.2	0.03	0.1	0.05	0.2
	Z = 0	0.86	0	0.01	0.04	0	90	0.1	0.2	0.03	0.1	0.05	0.2

## Data Availability

The dataset used during the current study is available from the corresponding author upon request.

## Abbreviations

2SLS: Two-stage least square
2SRI: Two-stage residual inclusion
AT: As-treated
CI: Confidence interval
DAG: Directed acyclic graph
IP-weighted PP: Inverse probability-weighted per-protocol
ITT: Intention-to-treat
IV: Instrumental variable
MSE: Mean squared error
NPCB: Nonparametric causal bound
PP: Per-protocol
RD: Risk difference
SE: Standard error
